# NanoporeDB: a structural resource of multimeric protein nanopores for single-molecule sensing

**DOI:** 10.1093/gigascience/giag076

**Published:** 2026-06-25

**Authors:** Yuqian Liu, Zidong Su, Wenzhen Yang, Denghui Li, Jiawen Zhang, Yuning Zhang, Tao Zeng, Yong Zhang, Yuxiang Li, Guangyi Fan, Kailong Ma, Shanshan Liu, Xun Xu, Yuliang Dong, Zongan Wang

**Affiliations:** College of Life Sciences, University of Chinese Academy of Sciences, Beijing 100049, China; BGI Research, Sanya 572025, China; BGI Research, Sanya 572025, China; School of Artificial Intelligence, University of Chinese Academy of Sciences, Beijing 100049, China; BGI Research, Shenzhen 518083, China; BGI Research, Sanya 572025, China; BGI Research, Qingdao 266555, China; BGI Research, Shenzhen 518083, China; BGI Hangzhou CycloneSEQ Technology Co., Ltd, Hangzhou 310030, China; BGI Research, Shenzhen 518083, China; BGI Hangzhou CycloneSEQ Technology Co., Ltd, Hangzhou 310030, China; BGI Research, Shenzhen 518083, China; BGI Hangzhou CycloneSEQ Technology Co., Ltd, Hangzhou 310030, China; BGI Research, Wuhan 430074, China; BGI Research, Wuhan 430074, China; BGI Research, Qingdao 266555, China; Shenzhen Key Laboratory of Bioenergy, BGI Research, Shenzhen 518083, China; BGI Research, Shenzhen 518083, China; BGI, Shenzhen 518083, China; Shenzhen Key Laboratory of Marine Biology Genomics, BGI Research, Shenzhen 518083, China; Institution of Deep-Sea Life Sciences, IDSSE-BGI, Hainan Deep-sea Technology Laboratory, Sanya 572000, China; College of Life Sciences, University of Chinese Academy of Sciences, Beijing 100049, China; BGI Research, Shenzhen 518083, China; State Key Laboratory of Genome and Multi-omics Technologies, BGI Research, Shenzhen 518083, China; Guangdong Provincial Key Laboratory of Genome Read and Write, BGI Research, Shenzhen 518083, China; BGI Research, Shenzhen 518083, China; BGI Hangzhou CycloneSEQ Technology Co., Ltd, Hangzhou 310030, China; State Key Laboratory of Genome and Multi-omics Technologies, BGI Research, Shenzhen 518083, China; Shenzhen Engineering Laboratory for Molecular Enzymology, BGI Research, Shenzhen 518083, China; BGI Research, Sanya 572025, China; BGI Research, Shenzhen 518083, China; State Key Laboratory of Genome and Multi-omics Technologies, BGI Research, Shenzhen 518083, China; Hainan Technology Innovation Center for Marine Biological Resources Utilization (Preparatory Period), BGI Research, Sanya 572025, China

**Keywords:** protein nanopore, single-molecule sensing, AlphaFold, protein structure prediction, transmembrane pore geometry

## Abstract

**Background:**

Protein nanopores are essential molecular gateways in biology and have inspired transformative technologies in biosensing and single-molecule sequencing. However, the discovery and engineering of novel nanopore scaffolds remains limited due to the scarcity of experimentally resolved pore structures.

**Results:**

Here, we present NanoporeDB, an open-access structural resource comprising about 7,000 high-confidence multimeric models across 4 representative pore types. Using a structure- and sequence-guided mining strategy, we identified candidate nanopores from large protein datasets, including the AlphaFold Protein Structure Database, UniRef90, and MGnify90, and generated high-confidence multimeric models using AlphaFold-Multimer and AlphaFold3. Collectively, these models represent a >170-fold expansion of the structurally annotated nanopore repertoire. Each model is further annotated with predicted membrane embedding, pore geometry, and constriction profiles, enabling structure-informed functional inference. NanoporeDB features an interactive web interface with 3D visualization and quantitative metrics.

**Conclusions:**

NanoporeDB provides the first comprehensive structural resource of multimeric protein nanopores with explicit membrane and pore annotations. This resource provides a structural gateway for advancing nanopore-based molecular sensing, precision diagnostics, and synthetic biology. NanoporeDB is publicly available at https://db.genomics.cn/nanopore.

## Graphical Abstract

**Graphical Abstract:**
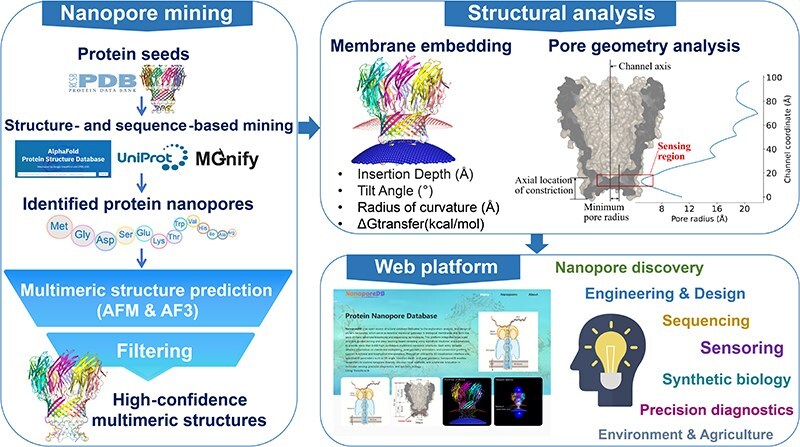

## Introduction

**Figure 1:**
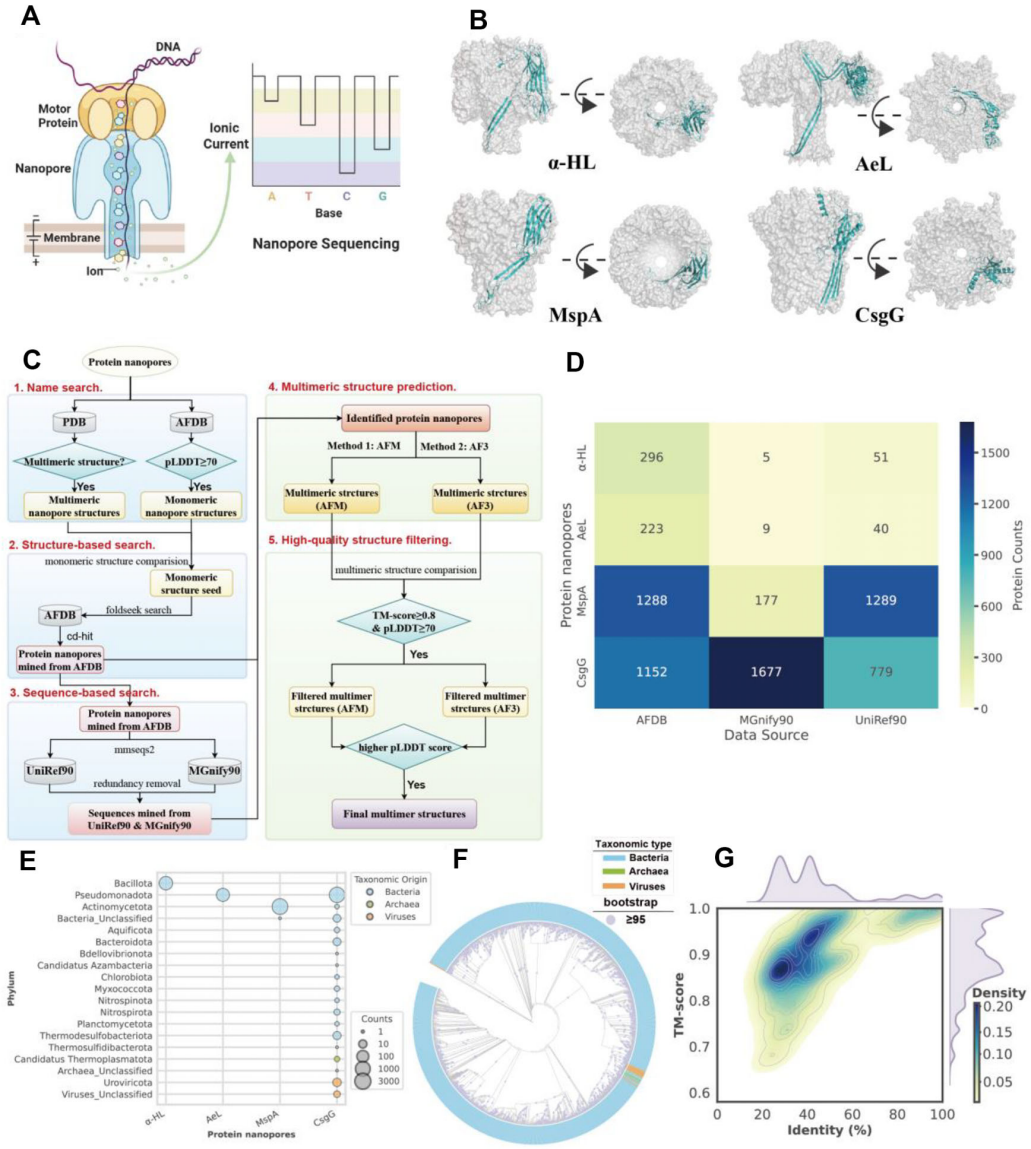
Structure- and sequence-based identification of protein nanopores. (A) Schematic illustration of nanopore structure and the principle of nanopore sequencing. (B) Structural representation of multimeric protein nanopores: α-HL (PDB: 7AHL), AeL (PDB: 9FM6), MspA (PDB: 1UUN), and CsgG (PDB: 4Q79). A monomeric chain of each nanopore is distinctly highlighted. (C) Overview of the nanopore mining workflow. (D) Source distribution of mined candidate protein nanopores. (E) Phylum-level distribution of annotated protein nanopores. The bubble chart displays protein nanopore counts across different phyla (*y*-axis) and nanopore types (*x*-axis). Bubble size corresponds to the number of proteins identified within each nanopore type. Bubbles are grouped by taxonomic domain (Bacteria, Archaea, and Viruses). (F) Phylogenetic tree of the identified CsgG proteins with taxonomic annotations. The annotation ring indicates the taxonomic origin of each sequence, and marked internal nodes denote branches with strong phylogenetic support (bootstrap ≥95). (G) Sequence-structure similarity landscape of candidate nanopores compared to representative PDB nanopore structures. The *x*-axis represents sequence identity, and the *y*-axis represents structural similarity (TM-score). The density map indicates the relative abundance of candidates across the sequence-structure similarity space.

Protein nanopores are transmembrane channels that mediate selective molecular transport, sensing, and signaling in living organisms [1]. Their biomimetic application, leveraging tunable pore geometries, has inspired transformative technologies in biosensing, sequencing, and macromolecular analysis [2]. Among these, nanopore sequencing has emerged as a powerful single-molecule technique that enables direct, label-free analysis of nucleic acids and proteins and beyond [1–3]. It relies on nanopores embedded in electrically insulating membranes (e.g., lipid bilayers) to form transmembrane channels with constriction zones, where voltage-driven translocation of analytes generates characteristic ionic current signals for real-time molecular identification and sequencing [4] (Fig. 1A). Owing to its unique advantages in long-read capability, real-time detection and minimal sample preparation, nanopore sequencing is transforming genomics and proteomics [3, 5–7]. Its versatility has extended applications from clinical diagnostics and epigenetic profiling to field-deployable biosensing [8–10]. The continued growth of nanopore-based technologies has also stimulated the development of specialized computational infrastructures for efficient management and access to large-scale sequencing datasets [11]. Over the past 3 decades, continual efforts have been devoted to engineering protein nanopores for improved stability, selectivity, and sensing resolution [4, 12, 13]. However, most rational designs remain limited to a few well-characterized protein nanopores, such as MspA and CsgG (Fig. 1B), due to the technical difficulty and high cost of resolving membrane protein structures, particularly the narrow constriction zones essential for function [12, 14].

The discovery and engineering of protein nanopores rely critically on assessing structural features such as pore geometry and membrane embedment. Recent breakthroughs in deep learning-based methods for protein structure prediction, such as AlphaFold [15–17], enable fast acquisition of protein structures accurate enough for downstream rational design. In addition, large databases of predicted protein structures, such as AlphaFold Protein Structure Database (AFDB) [18] and ESM Metagenomic Atlas [19], offer a unique opportunity for mining new candidates of nanopores with improved sequencing capability. Despite these advances, existing resources remain poorly suited for systematic nanopore discovery and analysis. Recent efforts have also highlighted the importance of automated data curation and annotation pipelines for large-scale structural biology resources, exemplified by CryoDataBot [20]. First, AFDB and ESM Metagenomic Atlas only provide predictions of single-chain proteins, whereas commercialized nanopores used for sequencing operate as large homomultimers, such as CsgG [21]. Because monomers undergo large conformational rearrangements upon assembly, direct prediction and analysis of the multimeric pore assemblies is advantageous in inspecting the interior of the pore lumen, especially the functional constriction site. Second, derivative databases of AFDB, such as TmAlphaFold [22], MembranomeX [23], ChannelsDB 2.0 [24], and AFTM [25], are focused primarily on transmembrane α-helical proteins but cover few β-barrel nanopores that dominate sensing applications.

To address the aforementioned limitations, we developed an open-access resource NanoporeDB, which is dedicated to systematically expanding the structurally annotated nanopore resource and provides an entry point for nanopore discovery and rational design. To construct NanoporeDB, we developed an integrated structure- and sequence-guided mining workflow that systematically identified homologs from AFDB [18], UniRef90 [26], and MGnify90 [27], using experimental structures from Protein Data Bank (PDB) [28] as references. Candidate homologs were subjected to multimeric structure prediction using a locally optimized AlphaFold-Multimer (AFM) [16] and AlphaFold3 (AF3) [17], and high-confidence structural models were retained after a stringent quality filtering for downstream analyses, including membrane embedding and pore geometry annotation. Compared to the few experimentally resolved multimeric structures currently available in the PDB (Supplementary Table S1), NanoporeDB contributes 6,986 high-confidence predicted models, expanding the structurally annotated nanopore repertoire by more than 170-fold. We anticipate that NanoporeDB will serve as a valuable resource to advance the exploration and engineering of nanopores, enabling next-generation innovations in molecular sensing, precision diagnostics, and synthetic biology.

## Results

### Structure- and sequence-guided workflow for protein nanopore mining

In this study, we developed a systematic mining workflow for a comprehensive encompassment of protein nanopores of interest (Fig. 1C). The pipeline consists of 5 consecutive steps: (1) name search. We retrieved protein nanopores by name from both PDB and AFDB. (2) Structure-based search. We aligned the predicted monomeric structures from AFDB against the experimentally resolved templates from PDB and saved only the highly similar structures (methods). The retained multimer templates and predicted monomers were combined as structural seeds to align search against the entire AFDB for candidate structures (methods). (3) Sequence-based search. All unique sequences from step 2 were used to search against UniRef90 and MGnify90. (4) Multimeric structure prediction. The candidate sequences obtained from steps 2 and 3 were merged and deduplicated for multimeric structure prediction by AFM and AF3, respectively. (5) High-quality structure filtration. All predicted models were aligned to the corresponding multimeric templates from PDB, whereas for each protein, only the model with higher pLDDT score was retained to avoid redundancy (methods).

We focused on four protein nanopores widely used in nanopore sequencing technologies on purpose, i.e., alpha-hemolysin (α-HL) [29], aerolysin (AeL) [30], MspA [31], and CsgG [32, 33]. Despite their significance, only a limited number of pore-like structures have been experimentally resolved for each protein type (Supplementary Table S1), highlighting the urgent need for expanding the protein nanopore repertoire. Using our systematic mining workflow, we identified a total of 6,986 candidate protein nanopores, including 352 α-HL, 272 AeL, 2,754 MspA, and 3,608 CsgG proteins (Fig. 1D, Supplementary Table S2), substantially broadening the available pool of protein nanopores for downstream structural and functional exploration. Among the nanopores with available taxonomic annotations from AFDB and UniRef90, the majority of α-HL, AeL, and MspA proteins were annotated to a single predominant phylum, reflecting a taxonomically concentrated distribution (Fig. 1E). In contrast, CsgG-like nanopores displayed a broader taxonomic span, with the majority assigned to bacteria (98.8%) and a smaller number originating from archaea and viruses (Fig. 1E). Notably, the archaeal and viral homologs are nested deeply within bacterial lineages (Fig. 1F), indicating potential horizontal gene transfer of CsgG-like nanopores across distant evolutionary lineages.

To evaluate the diversity of the candidate nanopores, we compared their structural and sequence similarities to experimentally resolved multimeric protein nanopores from the PDB (Fig. 1G, methods). Across all nanopore types, structural similarity consistently exceeded sequence similarity, indicating the conservation of nanopore structures despite considerable sequence divergence. These results demonstrate that our structure- and sequence-based mining workflow effectively detected remote homologs with conserved structural features but novel sequences, thereby expanding the protein nanopore pool beyond the reach of sequence-based strategies alone.

### Multimeric model prediction reveals confidence gains and conformational diversity

**Figure 2:**
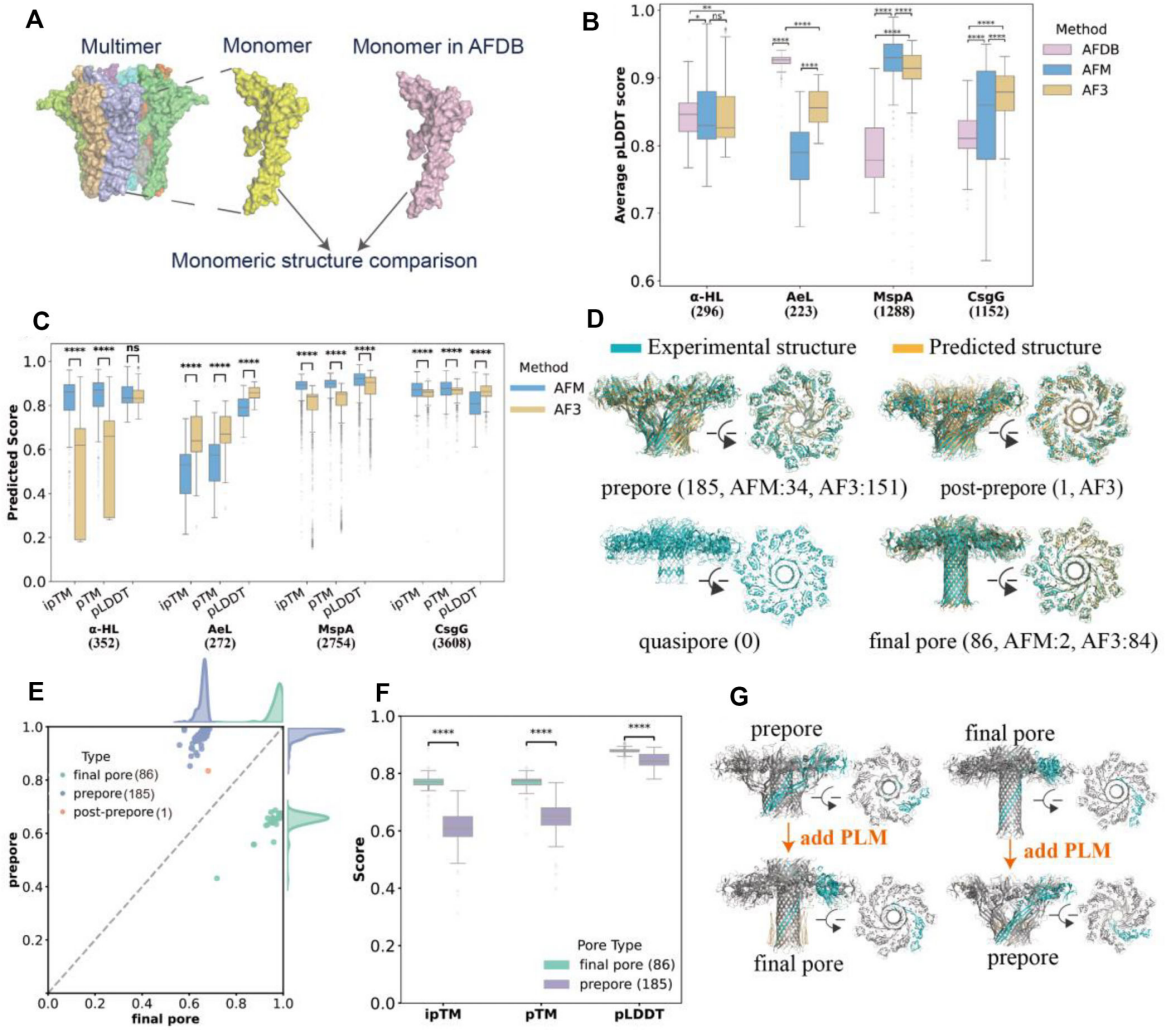
Evaluating predicted multimeric structures of identified protein nanopores. (A) Schematic of monomeric structure comparison. (B) Comparison of monomer-level average pLDDT scores across AFDB, AFM, and AF3 models for each type of nanopore. Models from different sources or predictors are distinguished as indicated in the plot legend. Numbers in parentheses indicate structures mined only from AFDB (Fig. 1D). (C) Comparison of multimeric structure prediction scores (average pLDDT, pTM, and ipTM) between AFM and AF3 models across all identified protein nanopores. Numbers in parentheses indicate structures mined from all resources (Fig. 1D). (D) Schematic representation of the 4 AeL conformations: prepore, post-prepore, quasipore, and final pore. Reference structures correspond to experimentally resolved states (prepore: 9FMX, post-prepore: 5JZW, quasipore: 5JZW, final pore: 9FML). Predicted AeL-like models with the highest similarity are aligned with the experimental structures. Numbers in parentheses indicate the models attributed to each conformational state and its predictor (Fig. 1C). (E) Conformational grouping of AeL-like models. Scatter plot shows TM-scores of predicted models relative to a prepore (*y*-axis) and a final pore (*x*-axis). (F) Multimeric self-confidence scores (ipTM, pTM, and pLDDT) for the AeL prepore and final pore conformations. (G) Conformational transitions observed after adding PLM molecules in joint structure prediction. A representative monomer chain is highlighted in each nanopore structure. In (B), (C), and (F), statistical significance of paired comparisons was calculated using a 2-sided Wilcoxon–Mann–Whitney *U*-test (*P < 0.05, **P < 0.01, ^****^P < 0.0001).

We assumed that predicting the multimers as a whole would improve the model quality of their constituent monomers compared to predicting the monomers alone when the inter-chain interactions were accounted for by AlphaFold. Therefore, we first compared the average pLDDT scores of the first chains of our predicted multimeric structures with their corresponding monomeric structures from AFDB (Fig. 2A). In general, the multimeric models predicted by AFM and AF3 had monomers of better quality than their counterparts in AFDB for MspA and CsgG, indicating improved local structural confidence (Fig. 2B). For MspA, models showed improvement with the median pLDDT scores of 0.78, 0.93, and 0.91 for AFDB, AFM, and AF3 models, respectively, in which AFM slightly outperformed AF3. Similarly, CsgG nanopores exhibited a progressive increase in confidence, with median pLDDT scores of 0.81, 0.86, and 0.88 in AFDB, AFM, and AF3, respectively. In contrast, for α-HL and AeL nanopores, multimer predictions (AFM and AF3) resulted in lower monomer confidence compared to AFDB models (Fig. 2B).

We then assessed the quality of the overall multimer models predicted by AFM and AF3 by comparing 3 structural metrics: ipTM measures the quality of interfaces between interacting monomers, pTM evaluates the general fold of the multimeric structures, and the average pLDDT scores indicate the confidence of local structures (Fig. 2C). In parallel to the above monomer comparisons, for MspA and CsgG nanopores, both methods yielded consistently high scores (>0.8) across all 3 metrics (Fig. 2C). In contrast, for α-HL, AFM models generally achieved higher ipTM and pTM scores than AF3, while both methods maintained high local confidence (pLDDT). Although α-HL is also known to adopt multiple conformational states, these variations are relatively subtle at the global structural level and are not further resolved here (Supplementary Fig. S1, Supplementary Table S4). For AeL nanopores, both methods exhibited markedly lower scores across all 3 metrics compared with the other pore types, and within this group AF3 significantly outperformed AFM (Fig. 2C).

The relatively lower confidence scores observed for AeL nanopores (Fig. 2B and C) prompted us to examine the potential impact of the conformational heterogeneity. During pore formation, the AeL proteins undergo a series of conformational changes: from prepore to final pore via 2 transient intermediate states, i.e., post-prepore and quasipore (Fig. 2D, Supplementary Table S1) [34]. This process begins with the oligomerization and formation of 2 stable concentric β-barrels (prepore); then, the protein morphs through a zipper-like formation and piston-like extension of the inner β-barrel (post-prepore and quasipore), and finally inserts into the lipid bilayer (final pore) [34]. Our workflow predominantly captured the prepore (185 of 272 models, 68%) and final pore (86 of 272 models, 32%) states based on the global structural similarity, whereas the post-prepore (1 model) and quasipore (0 model) states were underrepresented (Fig. 2D and E, methods). Further, we showed that the models adopting the final pore conformation consistently achieved high self-confidence scores, indicating that the lower overall confidence of AeL models primarily resulted from the dominance of prepore conformations, which were intrinsically more flexible and heterogeneous prior to membrane insertion (Fig. 2F, Supplementary Fig. S2).

Given the scarcity of these intermediate states in prediction, the subsequent analyses were focused on comparing the prepore and final pore states. Because our workflow would select the multimer model with higher self-confidence from the AFM and AF3 predictions (Fig. 1C), we noticed that both the prepore and the final pore conformations were represented mostly by AF3 models (Fig. 2D). We hence compared the 2 predictors. All AeL models predicted by either AFM or AF3 were aligned against the reference experimental structures of the prepore and final pore, respectively (Supplementary Fig. S3, Supplementary Table S3, methods). AFM models were strongly biased toward the prepore state, with about 80% exceeding a TM-score of 0.7 to the reference prepore structure and only a negligible fraction matching the reference final pore structures (Supplementary Fig. S3A, Supplementary Table S3). In contrast, AF3 predictions were distributed across both states, with 67% inclined to the prepore and 31% to the final pores (Supplementary Fig. S3B, Supplementary Table S3), underscoring AF3’s capability of broader conformational sampling.

We further examined whether the addition of membrane-mimicking components could alter the conformational switching (Fig. 2G). Hence, we introduced palmitic acid (PLM) as a lipid surrogate in AF3 predictions, which has a polar head and the longest lipophilic fatty tail available on AF3 online server. In total, 46 prepore-like AF3 models transitioned to the final pore state in the joint prediction of nanopore plus PLM, in which cases PLM molecules formed a bilayer, whereas 4 final pore-like models reverted to the prepore state with slightly reduced self-confidence (Fig. 2G, Supplementary Fig. S4A, Supplementary Table S5). Though predictions with PLM showed elevated pLDDT values, adding PLM did not improve ipTM and pTM scores (Supplementary Fig. S4B), indicating that introducing additional entities in AF3 prediction affected differently the local and global structure. Further, the observation that adding lipophilic entities in joint structure prediction biased protein conformation toward the transmembrane state (i.e., the final pore state) naturally led us to postulate the opposite scenario of adding hydrophilic entities. Thus, we replaced PLM molecules with potassium and chloride ions (Supplementary Fig. S5). As expected, we observed 84 models with conformational changes, predominantly from the final pore state to the prepore state (*n* = 64), with transitions summarized in Supplementary Table S5.

### Structural annotation reveals membrane compatibility and pore geometry diversity

**Figure 3:**
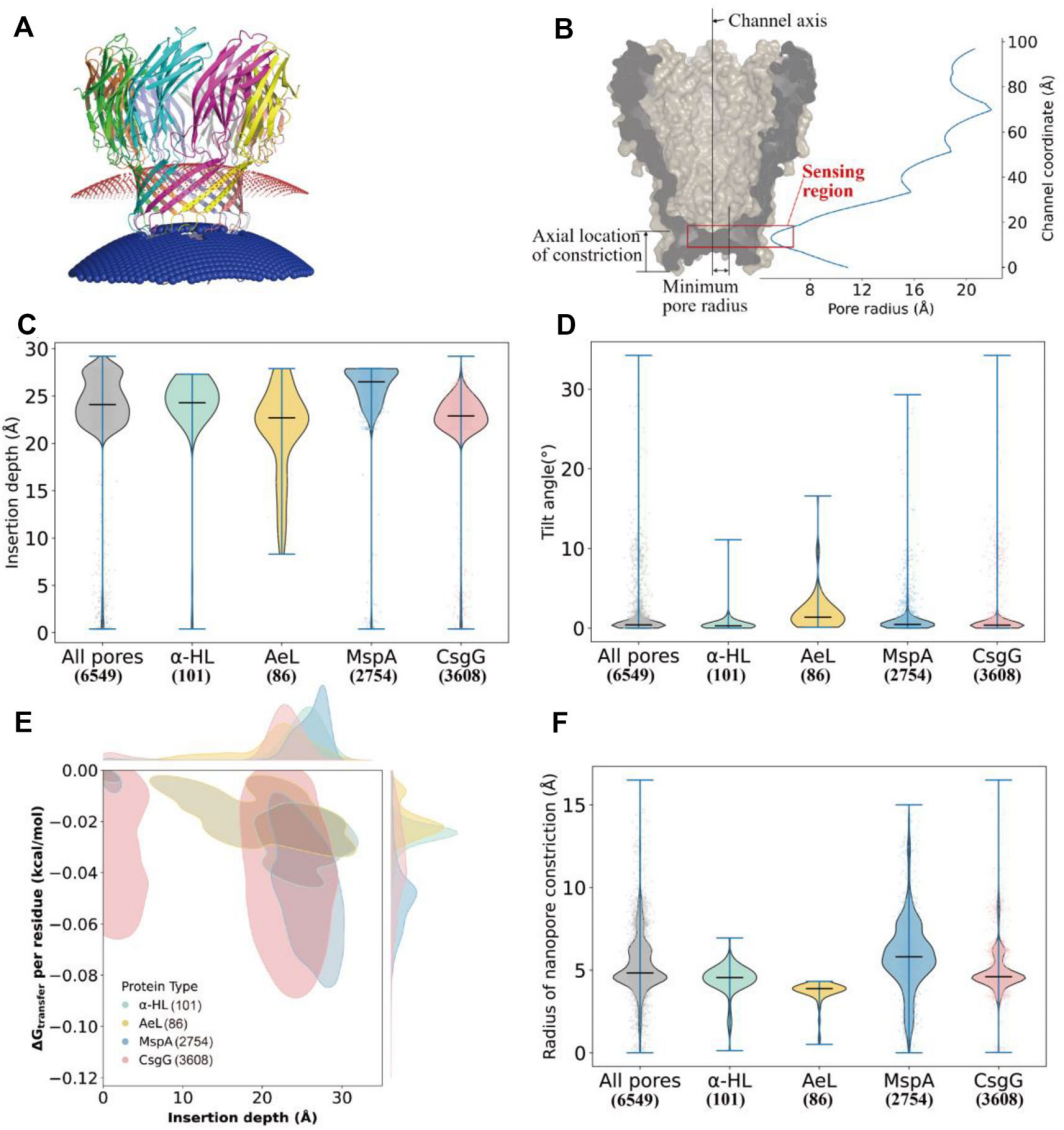
Membrane embedding and structural features analysis of candidate protein nanopores. (A) Example of predicted nanopore embedding. The upper and lower boundary markers indicate the outer and inner membrane surfaces, respectively. (B) Example of the minimum pore radius and its corresponding axial location along the nanopore channel. Representative profile of pore radius along the channel axis for an MspA-like nanopore (AFDB ID: A0A064C8×8). (C) Violin plot of predicted insertion depth (Å) for nanopore candidates. (D) Violin plot of predicted tilt angles (°) relative to the membrane normal (methods). (E) Density plot of transfer free energy per residue for nanopore candidates. (F) Violin plot of predicted minimum pore radius values (Å) across nanopore candidates.

To facilitate the application of candidate protein nanopores for single-molecule sensing, we performed in-depth structural analysis, including membrane embedding and pore constriction analysis (Fig. 3A and B). The latter was focused on computing pore radius and the axial location along the pore channel (Fig. 3B). We first computed the insertion and orientation of each multimeric complex within a model lipid bilayer (1,2-dioleoyl-sn-glycero-3-phosphocholine, i.e., DOPC) using PPM 3.0 (Positioning of Proteins in Membranes) [35]. For each model, we computed its key structural parameters, including the insertion depth and the tilt angle of the pore axis relative to the membrane normal. It is noted that we only included AeL-like candidates exhibiting the final pore conformations in membrane embedding analysis (Fig. 2D), because the prepore state is not expected to enter the membrane [34]. Because nearly 68% of AeL-like models were prepore-like, the conformation-based filtering was essential to evaluate nanopore candidates. Nonetheless, being in the final pore state did not guarantee membrane insertion (Supplementary Figs S6B and S7A). This cautions against relying on a single criterion in candidate assessment.

Across all analyzed complexes, the predicted nanopores were embedded in lipid bilayers with a median insertion depth of 23.9 Å (Fig. 3C). The central pore axes were uniformly oriented perpendicular to the local membrane surface with a median tilt angle of 0.4° (Fig. 3D) with a few exceptions (Supplementary Fig. S7). Nanopore-specific variations were observed: α-HL and MspA-like pores showed deeper insertions (median ≈ 24 Å and ≈ 26 Å, respectively), whereas AeL- and CsgG-like pores exhibited shallower embeddings (median ≈ 23 Å), reflecting pore-specific hydrophobic matching. A minority of all models (3.3%) displayed anomalous shallow insertion depths (<20 Å) (Fig. 3C, Supplementary Fig. S6). For MspA-like pores, such cases likely reflect possible multiple conformations similar to that of AeL (Supplementary Fig. S6C). For CsgG-like pores, those anomalies are associated with the absence of the native N-terminal membrane anchor (Supplementary Fig. S6D), which was previously shown to be essential for bilayer insertion [32]. Re-prediction of these cases using full-length sequences restored correct membrane embedding in the majority of instances (339 of 425 models, 79.8%), indicating that the effect arises from missing anchoring elements rather than incorrect folding of the pore-forming core. The remaining cases retained atypical membrane positioning despite highly similar pore-forming core structures, indicating that the observed deviations primarily affected membrane annotation rather than nanopore architecture itself (Supplementary Note 1). Importantly, structural comparisons demonstrated that the pore-forming cores remained highly consistent between trimmed and full-length predictions (Supplementary Figs S8–S10, Supplementary Note 1). This highlights a trade-off between computational efficiency and accurate membrane positioning, while confirming the robustness of the nanopore core architecture. From the perspective of energetics, the nanopore-specific differences were shown in the distribution of transfer free energy per residue: pores with deeper insertion generally had more favorable insertion energies (Fig. 3E). MspA exhibited the greatest mean insertion depth (Supplementary Fig. S11), which more closely matched the hydrophobic thickness of DOPC bilayer [36], and thereby minimized the hydrophobic mismatch to obtain the most favorable transfer energy (Fig. 3E).

To further evaluate the predicted nanopore assemblies, we performed complementary analyses of interface confidence and dynamical stability. As an interface-focused confidence metric, mpDockQ showed family-dependent distributions (Supplementary Table S8), with median values ranging from 0.263 for α-HL and AeL to 0.344 for CsgG. Because α-HL and AeL datasets contained multiple conformational states, we additionally analyzed pore-state subsets separately, which exhibited distinct mpDockQ distributions from the corresponding full datasets. We note that mpDockQ was used descriptively and not as a filtering criterion because no universally accepted threshold has been established for multimeric assemblies. Consistent with these distributions, mpDockQ and ipTM exhibited similar rank-order trends across nanopore families (overall Spearman ρ = 0.77; Supplementary Fig. S12). In addition, 100 ns all-atom molecular dynamics (MD) simulations were performed for 4 representative nanopores from α-HL, AeL, MspA, and CsgG families (Supplementary Fig. S13). These simulations are intended as illustrative case studies rather than a comprehensive assessment of the entire database. In all 4 cases, the pore-forming β-barrel domains remained structurally stable throughout the trajectories, exhibiting relatively low RMSD fluctuations despite larger motions observed for some full assemblies (Supplementary Fig. S14, methods). Consistently, pore radius profiles calculated along the channel axis exhibited only limited fluctuations without significant lumen collapse during the simulations (Supplementary Fig. S15). We further note that the AeL simulation represents a single pore-state model and should not be interpreted as representative of the broader prepore-dominated AeL population described above.

To further gauge the structural suitability of the candidate protein nanopores for potential analyte translocation, we analyzed their pore geometries. We calculated the pore radius along the central channel axis to identify the location and width of the narrowest constriction zone (Fig. 3B and F), which are critical determinants for sensing resolution and analyte compatibility [37]. The predicted MspA-like model displayed consistent features with the canonical MspA nanopores [38], including a minimal pore radius of ∼5 Å positioned at ∼13 Å above the β-barrel terminus along the channel axis (Fig. 3B). Narrow constriction is a known characteristic to enhance single-molecule signal discrimination during translocation [31, 39]. In general, the predicted minimum pore radii exhibited a wide range (0–16 Å), with the vast majority (>93%) of structures falling within the 3–10 Å range (Fig. 3F). This size range permits the translocation of single-stranded DNA and small peptides [31, 40], suggesting the potential for further engineering toward single-molecule sensing applications. While the majority of candidates held promise for sensing applications akin to current nanopore technologies, the existence of pores with both smaller and larger constrictions hinted at a wider functional potential that merits further investigation (Supplementary Fig. S16).

### NanoporeDB: a web-based resource for structural profiling of protein nanopores

**Figure 4:**
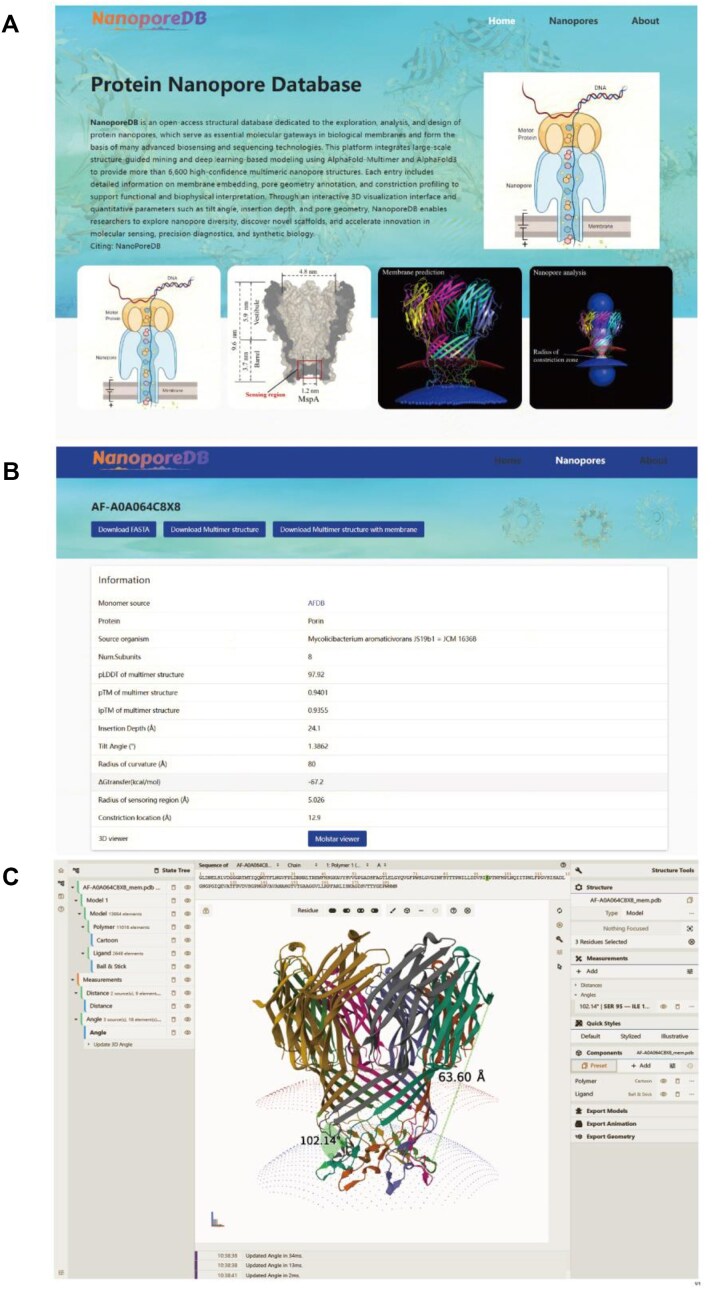
Web interface and data visualization of NanoporeDB. (A) Homepage of the NanoporeDB website. (B) The webpage of a deposited nanopore candidate detailing the information for individual protein nanopores, including sequence, structure, and structure annotations. (C) Example of the 3D visualization UI of a membrane-embedded protein nanopore structure.

To facilitate the discovery and structural evaluation of protein nanopore candidates, we developed an interactive web platform NanoporeDB (Fig. 4A). For each entry, NanoporeDB provides the multimeric assembly predicted by either AFM or AF3, which can be freely downloaded on the entry page (Fig. 4B). Structural features relevant to nanopore sensing, including the minimum pore radius and its corresponding axial position relative to the β-barrel terminus, insertion depth, tilt angle, and transfer free energy, are displayed as well (Fig. 4B). Examining these parameters (e.g., minimum pore radius <6 Å, tilt angle <10°) can allow users to readily identify nanopore candidates that meet the dimensional requirements for the intended sensing or sequencing applications.

In addition, we implemented an interactive “3D viewer” user interface (UI) enabling flexible online inspection of pore structure for each entry (Fig. 4C). Users can conveniently explore the multimeric nanopore structures at multiple levels, from individual atoms and residues to complete chains. Beyond flexible selection and visualization, the UI supports precise structural measurements such as the distances between residue pairs, bond lengths, and dihedral angles among selected residues (Fig. 4C). The integrated visualization, along with downloadable model files and detailed structural metrics, enables efficient screening and rational design of nanopore-based biosensing and sequencing applications.

## Discussion

Traditional sequence-based mining approaches are often limited in detecting remote homologs, as evolutionary divergence rapidly erodes sequence similarity while leaving structural features comparatively conserved. Recent studies have demonstrated that structure-based or structure-informed strategies can substantially expand the discovery of novel protein families, such as novel deaminases identified through structure clustering [41] and TIGR-Tas systems uncovered by integrating predicted structures with sequence features [42]. Consistent with these observations, our results show that structural similarity among nanopore candidates was consistently higher than sequence similarity (Fig. 1G), demonstrating the effectiveness of structure-guided mining in identifying novel protein nanopores and extending the protein nanopores beyond the reach of sequence-based approaches.

Among the nanopore candidates, α-HL and AeL-like nanopores are largely associated with pathogenic bacterial lineages, and MspA is primarily confined to the Actinomycetota (Fig. 1E). In comparison, CsgG-like proteins exhibit a markedly broader taxonomic distribution compared to α-HL, AeL, and MspA nanopores, with homologs detected across multiple bacterial phyla, as well as in archaea and viruses. This widespread occurrence likely reflects the conservative role of CsgG as the outer-membrane channel of the curli secretion system, which is essential for biofilm formation and environmental adaptation in many Gram-negative bacteria [43, 44]. The wider phylogenetic distribution of CsgG homologs suggests possible horizontal gene transfer, as secretion-system components are known to be prone to horizontal transfer [45]. For instance, metagenomic surveys of seawater phages showed strong enrichment of adhesion-related genes with CsgG being the most abundant [46]. Given that our structural mining strategy relies on available PDB templates, evolutionary proximity to known structures may influence detection sensitivity, particularly for nanopores with restricted phylogenetic distributions. Therefore, the observed lineage enrichment should be interpreted with this methodological consideration in mind. At the same time, the distinct taxonomic distributions observed among different nanopore types, particularly the broader, cross-phylum representation of CsgG homologs, indicate that the distributions are unlikely to arise solely from templates origin and may also reflect underlying biological and evolutionary characteristics of these nanopores (Fig. 1E and F, Supplementary Table S5).

Our comparative analysis revealed that for protein nanopores the monomeric subunits extracted from AFM- and AF3-predicted multimeric structures generally exhibited higher average pLDDT scores than their monomeric counterparts from the AFDB (Fig. 2B). This improvement in local structure prediction demonstrates that the explicit incorporation of inter-chain interactions during multimeric folding can refine model quality of the constituent monomers, which may result from resolving conformational ambiguities at multimeric interfaces and within pore-lining regions that remain poorly captured in isolated monomer predictions. Admittedly, more reliable interface-specific metrics may offer better comparisons of predicted complex models [47–51]. Consistent observations across α-HL, MspA, and CsgG-like nanopores emphasize the necessity of multimeric modeling for protein nanopores (Fig. 2B). Moreover, modeling the entire multimer offers the particular advantage of illuminating regions that are often unresolved in experimental structures. For example, the secretin GspD of the type II secretion system is a huge pentadecameric nanopore [52] and has been reported as a competent next-generation candidate for DNA sequencing applications [53, 54]. Multimer structure prediction can provide valuable hypotheses [55] of the unresolved key linkers in the central and cap gates of GspD, which are critical for its sensing ability [52].

On the other hand, AeL-like proteins represented an informative exception that merits further consideration (Fig. 2). First, AFM predictions were strongly biased toward the prepore state (Supplementary Fig. S3A), whereas AF3 models were distributed in both conformational states in a more balanced manner (Supplementary Fig. S3B), indicating AF3’s advantageous performance in conformational sampling for certain proteins with multiple intermediate states. But AF3 is known for its limited ability in distinguishing conformational switching [17, 56]. Besides, neither approach yielded a better monomer in predicting the complex structure, in either prepore or final pore state, than the monomeric model from AFDB, which was directly predicted by AF2 (Supplementary Fig. S2A). These reflect the intrinsic challenge in predicting the multimeric structure of protein nanopore, especially when it undergoes large conformational transitions during the embedment into membrane [34, 57]. The long-tailed distributions in ipTM and pTM scores of MspA and CsgG (Fig. 2C) likely suggest the existence of prepore intermediates yet to be captured by experiment (Supplementary Fig. S5C left, S5D left). Similar prepore-like conformations of α-HL, which are supported experimentally [57], were also identified in the updated analysis incorporating recently resolved intermediate-state structures. However, because subsequent analyses in this study focused primarily on fully formed transmembrane pores and pore-geometry characterization, these conformations were not emphasized in the downstream structural analyses (Fig. 1C). Second, incorporation of membrane-mimicking components in AF3 effectively modulated the outcomes: PLM promoted conformational shifts toward final pores and hence rescued the membrane embedding that 65 out of 127 (51.1%) AeL-like final pores were able to be inserted into bilayer correctly (Supplementary Table S5, second column), among which there were 23 prepores switching to the final pore state, while there were only 20 out of 84 (23.8%) final pores that were able to embed correctly without PLM (Supplementary Table S5, first column). Similar co-folding outcomes were seen in 26 out of 32 MspA and 19 out of 41 CsgG models (Supplementary Fig. S16, Supplementary Table S2, methods). Still, this co-folding strategy does not promise better protein models in general (Supplementary Figs S3B and S4). Though previous works demonstrated that AF3 did not learn the physics but memorized ligand–protein interactions instead [56, 58, 59], our observations suggest that environmental surrogates can sway conformational sampling and hence produce desirable models. In general, accurately predicting multiple conformations of assemblies with morphing domains requires physics-informed structure prediction methods, possibly in combination with emulation of protein equilibrium ensembles [60].

Together, these findings establish NanoporeDB as a valuable resource for accelerating nanopore discovery and design in biosensing, sequencing, and diagnostic applications. Nevertheless, several limitations of our current version should be acknowledged. First, although AlphaFold-based models of protein nanopores generally exhibit high quality, experimental validation is essential to confirm their ability to capture functional conformations and properties relevant to desired applications, such as nanopore sensing. Second, the structural analysis of pore geometry depends critically on robust determination of the channel axis, which may be affected by local asymmetry or structural flexibility in some multimeric assemblies. Thus, manual curation of the pore axis served as a complement to automatic computing tools, such as HOLE [61], to ensure reliable radius profiling across the diverse nanopore structures. Third, membrane embedding was modeled under simplified conditions, assuming a static protein conformation and a homogeneous lipid bilayer. In reality, biological membranes are compositionally heterogeneous and dynamically fluid, with lipid–protein interactions, lateral pressure variations, and local curvature, which affect pore orientation, stability, and function [62, 63]. Fourth, our multimer prediction presumed the same stoichiometry as the experimental structure of given protein nanopore. However, isoforms of transmembrane channels are known to form diverse oligomeric assemblies [64]. Therefore, some of our predicted models with small pore radii may in fact assemble with more monomers, or vice versa. Fifth, the current version of NanoporeDB merely encompasses a limited set of nanopores, primarily focusing on representative and well-characterized nanopore types with commercial applications. The future release of NanoporeDB will expand the scope to include additional pore types such as GspD [53, 54].

## Methods

### Data collection

To identify protein nanopores on a large scale, we collected data from several high-quality structural and sequence databases. Experimentally determined protein structures were retrieved from the PDB (available as of March 2025) [28], while predicted protein structures were obtained from the AFDB v4 (available as of March 2024) [18]. Only AFDB monomeric models with high confidence (average pLDDT ≥ 70, as residues with pLDDT values above 70 are generally considered to possess reliable local backbone geometry [15, 18]) were considered. In addition to structural data, we used UniRef90 (version: November 2024) [26] and full-length protein sequences from MGnify90 (version: April 2024) [27] as comprehensive protein sequence databases for downstream homology searches.

### Protein nanopore mining workflow

To systematically identify and model protein nanopores from large-scale structure and sequence databases, we established a 5-step computational workflow (Fig. 1C).

1. Name-based retrieval. Representative nanopore proteins, including α-hemolysin (α-HL), aerolysin (AeL), MspA, and CsgG, were first retrieved by name from the PDB and AFDB v4 [18]. Only experimentally resolved multimeric pore-forming assemblies were retained to ensure structural relevance. These served as initial structural templates for subsequent searches.
2. Structure-based search. Predicted monomeric structures from AFDB were aligned to each experimentally resolved nanopore monomer from PDB using the *easy-search* module of Foldseek (v9.427df8a) [65]. Only hits with TM-score ≥ 0.8 were retained to ensure high structural confidence. (Previous studies have shown that TM-score > 0.5 generally indicates proteins sharing the same overall fold, whereas higher TM-scores correspond to increasingly stringent structural similarity [66]. We therefore adopted a conservative threshold of TM-score ≥ 0.8 to ensure high global structural consistency between predicted nanopore candidates and experimentally resolved nanopore assemblies.) The retained AFDB monomers and PDB templates were then merged as structural seeds for an expanded search across the entire AFDB database, enabling the identification of potential homologous nanopores with conserved structures but divergent sequences.
3. Sequence-based search. All unique sequences obtained from the structure-based search were further used as queries against UniRef90 and MGnify90 databases using MMseqs2 (commit: 0b27c9d7d7757f9530f2efab14d246d268849925) [67] (*-c 0.9 -e 1e-4*). This step expanded the nanopore candidates by identifying distant homologs sharing sequence or structural similarity.
4. Multimeric structure prediction. Candidate sequences from steps 2 and 3 were merged and deduplicated. Multimeric structures were predicted using AFM (locally optimized implementation; details provided below) [16] and AF3 web server (released 18 March 2025) [17]. Predictions were performed under identical subunit stoichiometries as the corresponding experimental templates.
5. Quality filtering and redundancy removal. All predicted multimeric structures were aligned to their corresponding PDB templates using US-align (v20241108) [68]. Only models with TM-score ≥ 0.8 were retained. For cases with multiple predictions of the same sequence, the structure with the highest pLDDT score was selected as the representative nanopore model.

### Monomeric structure comparison

To assess the structural similarity of predicted monomeric units, we used Foldseek easy-search (-c 0.7 -e 0.01 –tmscore-threshold 0.8 –alignment-type 1 –format-output query,target,alntmscore,qtmscore,ttmscore,qcov,tcov,lddt,prob). For each pairwise comparison, monomeric models with either qtmscore or ttmscore > 0.8 were retained as high-confidence structural matches.

### Multimer structure prediction

To predict the multimeric structures of candidate protein nanopores identified through sequence- and structure-based mining, we employed both AFM and AF3. Prior to structure prediction, we applied a truncation strategy to improve prediction accuracy and reduce computational load. Specifically, each candidate sequence was aligned to structurally resolved protein nanopores in the PDB using BLAST+ 2.16.0 [69], and the closest homolog (with the highest sequence identity) was selected as the reference. Residues extending beyond the N- and C-terminal boundaries of the reference structure were removed to eliminate potentially disordered or irrelevant regions that could interfere with accurate structure prediction. The refined sequences were then subjected to AFM and AF3 for multimeric structure prediction. Both methods provided accurate and efficient structural predictions across different types of nanopores with diverse oligomeric architectures. We noted that longer sequences required substantially greater computational time and memory, highlighting the need for efficient sequence preprocessing prior to inference.

AFM was implemented as a GPU memory-optimized variant of AFM, capable of handling input sequences up to ∼8,200 amino acids. Instead of directly modifying the original JAX-based AFM, which provides limited flexibility for GPU memory management, we developed our implementation based on Uni-Fold v2.2.0 [70]. Uni-Fold is an open-source PyTorch re-implementation of AlphaFold2 that maintains full compatibility with the official model parameters and supports both training and inference. It introduces a 2-dimensional blocking strategy within the Triangular Multiplication module to reduce peak GPU memory usage, providing a more flexible and extensible foundation for large-scale structure prediction. Using the converted parameters *multimer_model_1* (released 6 December 2022) [16], structural inference was performed on NVIDIA A100 GPUs (80 GB). To overcome the default memory limitations of Uni-Fold, which typically fails for sequences longer than ∼4,300 residues, we implemented a targeted memory optimization strategy. Redundant intermediate tensors were discarded, temporarily unused variables were offloaded to CPU memory and reloaded dynamically when required, and inference was conducted in bfloat16 precision to further reduce memory overhead. These enhancements collectively enabled stable prediction of multimeric complexes up to ∼8,200 residues, thereby facilitating the accurate modeling of large assemblies such as protein nanopores. The inference configuration used in this study was *model_name* = multimer_af2_v3, *base_seed* = 42, *num_ensembles* = 1, *max_recycling_iters* = 3, *bf16* = True.

In parallel, multimeric structures were also predicted using AF3 via the official AF3 Server (released 18 March 2025) [17]. As with AFM, input sequences were first truncated based on their closest structural homologs in the PDB. The number of protomers in each assembly was determined based on the canonical oligomeric state of the corresponding nanopore type (e.g., heptamer for α-HL and AeL, octamer for MspA, nonamer for CsgG). Each processed sequence of the candidate was submitted to the AF3 server, which generated 5 predicted models. The model with the highest *ranking_score* was selected for downstream analyses. This strategy ensured that only the most reliable multimeric assemblies were retained for subsequent structural comparisons, pore annotation, and functional interpretation.

### Multimeric structure comparison

To assess the global similarity of predicted multimeric assemblies, we used US-align [68] (*-mol prot -mm 1 -ter 1 -outfmt 2*) to align each predicted multimeric structure against experimentally resolved multimeric structures from the PDB. Models exhibiting either *qtmscore* or *ttmscore* > 0.8 were retained, ensuring that only high-confidence matches with consistent quaternary topologies were considered. The retained multimeric structures were further analyzed for membrane embedding and pore geometry.

### Conformation assignment of AeL-like models

We compared each predicted multimeric model with all experimentally resolved structures (Supplementary Tables S1 and S3) using US-align [68] (*-mol prot -mm 1 -ter 1 -outfmt 2*). Each model was assigned to the conformation yielding the highest *qtmscore* if *qtmscore* ≥ 0.7. Models with *qtmscore* < 0.7 were assigned as “other,” indicating a potential intermediate or uncharacterized conformation.

### Conformation assignment of α-HL-like models

Unlike AeL-like models, the experimental conformational states of α-HL exhibit exceptionally high global structural similarity, rendering global alignment metrics insensitive for state assignment. To objectively classify the α-HL-like models, we employed a local geometric metric termed the “Cap-to-Tip Distance.” This was defined as the Euclidean distance between the Cα atoms of 2 structural anchors: a rigid site in the cap domain (equivalent to Lys50 in 7AHL) and the flexible apex of the pore-forming stem (equivalent to Lys131 in 7AHL). These anchor positions were mapped to each candidate via pairwise sequence alignment with the reference α-HL sequence. Based on the distance ranges derived from experimental structures (e.g., ∼18 Å for prepore and ∼96 Å for pore), models were categorized into 3 states: “prepore-like” for distances <40 Å, “late-prepore-like” for distances between 40 and 85 Å, and “pore-like” for distances ≥85 Å.

To evaluate potential template-induced bias, each candidate was traced back to the conformational state of its primary search template. The “template state” (pore vs. prepore) was assigned based on the PDB structure that yielded the highest alignment score during the initial structure-based search stage. The independence of the final prediction was verified by comparing the distance distributions across different template-state groups.

### Phylogenetic analysis of identified CsgG proteins

Protein sequences of identified CsgG nanopores with available taxonomic annotations (bacteria, archaea, and viruses) were aligned using Clustal Omega (v1.2.4) [71] with default parameters. To root the phylogenetic tree, one homologous protein sequence (UniProtKB: Q1RD49) was included as an outgroup. The multiple sequence alignment was refined by removing poorly aligned positions and gaps using trimAl (v1.5) [72] with the -automated1 setting, producing a high-quality alignment. Phylogenetic reconstruction was performed with IQ-TREE 3 (v3.0.1) (*-m MFP -B 1000 -alrt 1000*). The resulting Newick tree was visualized and annotated in iTOL (v7.2.2) [73].

### Multimer interface assessment

To provide an additional interface-focused confidence assessment, we calculated mpDockQ scores for all predicted multimeric assemblies using the parameters reported in the original study [74]. mpDockQ combines the number of inter-chain contacts with the average pLDDT of interface residues through a sigmoid-based transformation and was developed for evaluating AlphaFold-predicted multimeric complexes. Because no generally accepted quality threshold has been established for mpDockQ in multimeric assemblies, the metric was used descriptively and was not applied as a filtering criterion. Pearson and Spearman correlation coefficients were calculated to assess the relationship between mpDockQ and AlphaFold-derived ipTM scores. Per-family and per-conformation distributions were summarized using medians and interquartile ranges and reported in Supplementary Table S8.

### MD simulations and stability analysis

To provide illustrative examples of dynamical behavior, rather than a comprehensive assessment of all predicted nanopores, 100 ns all-atom MD simulations were performed for one representative model from each nanopore type: α-HL (UniRef90_UPI000BF7BC04), AeL (UniRef90_UPI000B8E5A08), MspA (MGYP001806187341), and CsgG (UniRef90_X1AGU0).

1. System preparation and equilibrium. Simulation systems were constructed using the CHARMM-GUI Membrane Builder [75]. Each multimeric assembly was embedded into a pre-equilibrated DOPC lipid bilayer and centered in a rectangular box with a minimum clearance of 15 Å between the protein surface and the box boundaries. The systems were solvated with TIP3P water molecules and neutralized with 0.15 M KCl to mimic physiological ionic strength. The CHARMM36m force field [76] was employed for all protein, lipid, and ion components. CHARMM-GUI provided the optimized GROMACS input files (steps 6.0–6.6) for energy minimization and equilibration.
  All simulations were conducted using the GROMACS (v2026.0) [77]. Energy minimization was first performed using the steepest descent algorithm for 5,000 steps (or until the maximum force 1,000 kJ/mol/nm). Subsequently, the systems underwent a standardized 6-step equilibration protocol recommended by CHARMM-GUI, which included (i) a 250 ps NVT ensemble at 303.15 K using the v-rescale thermostat; and (ii) a total of 1.625 ns NPT ensemble at 1 bar using the C-rescale barostat with a semi-isotropic coupling scheme. During these stages, position and dihedral restraints on the protein and lipid headgroups were gradually released (from 4,000 to 50 kJ/mol/nm²) to ensure smooth relaxation of the system. Long-range electrostatic interactions were treated using the Particle Mesh Ewald method with a 1.2 nm cutoff.
2. Production MD. Following equilibration, unrestrained production MD simulations (step 7) were performed in the NPT ensemble for 100 ns with a time step of 2 fs. The temperature (303.15 K) and pressure (1.0 bar) were maintained using the v-rescale thermostat and C-rescale barostat, respectively. Short-range electrostatic and van der Waals interactions were calculated with a 1.2 nm cutoff, with the latter utilizing a force-switch modifier from 1.0 to 1.2 nm. All bonds involving hydrogen atoms were constrained using the LINCS algorithm. Trajectories were recorded every 20 ps for downstream analysis.
3. Trajectory analysis. Structural stability was quantified by calculating the root mean square deviation (RMSD) of the backbone atoms for both the entire multimeric complex and the core pore-forming β-barrel domain. To assess the functional consistency of the pore lumen, 100 structural snapshots were extracted from the 100 ns trajectory (at 1 ns intervals). For each snapshot, the pore radius profile along the channel axis was calculated using a systematic geometric protocol with the HOLE program [61]. To characterize the steady-state dimensions of the nanopores, statistical analysis (mean and standard deviation) of the radius profiles was performed using the final 50 ns of the simulation.

### Multimer structure prediction with PLM and ionic environments

Multimeric nanopore structures were predicted using AF3 in protein–ligand–multimer mode with ∼40 palmitic acid (PLM) molecules per model to mimic lipid surroundings. Control runs were performed with 10 K+ and 10 Cl⁻ ions to assess environmental effects on assembly formation. Model confidence was evaluated using pLDDT, pTM, and ipTM scores.

### Membrane embedding prediction

Membrane insertion prediction was performed for all high-confidence multimeric nanopore structures using PPM 3.0 [35]. We selected 1,2-dioleoyl-sn-glycero-3-phosphocholine (i.e., DOPC) as the reference lipid bilayer, which closely approximates the physicochemical properties of membranes typically used in nanopore sequencing experiments. PPM 3.0 automatically determines the energetically favorable membrane orientation by minimizing the transfer free energy from aqueous to membrane environments. For each structure, PPM 3.0 selected either a planar or curved bilayer model, depending on the structural curvature and energy landscape. The membrane representation generated by PPM 3.0 corresponds to a continuous implicit hydrophobic slab rather than explicit lipid molecules. Therefore, for pore-forming proteins, the membrane boundary may visually overlap with the solvent-accessible pore lumen in structural visualization. This representation does not indicate physical lipid penetration into the channel interior. The membrane slab annotations were used only for membrane positioning and orientation assessment and were not included in downstream pore-geometry calculations. The output included detailed structural parameters such as insertion depth, tilt angle, and transfer free energy. These predicted parameters may exhibit minor version dependence, as updates to energy functions or implicit membrane models across different PPM versions can influence the precise orientation and insertion values. However, the overall assessment of membrane insertion and pore orientation remains robust. These parameters were used to assess compatibility with lipid bilayers and to support downstream comparative analyses. Models exhibiting atypical membrane positioning after full-length re-prediction were retained in NanoporeDB because their pore-forming core structures remained intact. These entries were annotated in NanoporeDB as having atypical membrane embedding, and membrane-related parameters should be interpreted with caution.

### Membrane embedding evaluation

To assess the orientation and membrane compatibility of predicted nanopores, we evaluated their membrane embedding geometry using the PPM 3.0 predicted models. For each multimeric structure, the nanopore channel axis was defined as described in the “Pore radius calculation.” The planar (or curved) bilayer surface generated by PPM 3.0 was then used to derive its local normal vector. The tilt angle was calculated as the acute angle between the nanopore channel axis and the membrane normal, representing the inclination of the pore relative to the membrane plane. A nanopore was defined as *“able to embed”* if it had residues located on both sides of the membrane bilayer, indicating successful transmembrane insertion. Among these, structures showing a tilt angle of ≤10° were further classified as “*able to embed correctly*,” reflecting near-perpendicular alignment to the bilayer. These metrics allowed us to assess membrane compatibility and inform downstream analyses of pore geometry and structural comparability.

### Pore radius calculation

To characterize the internal pore architecture, we employed HOLE (v2016.8.7) [61] to compute the radius profile along each nanopore’s channel axis. Given that HOLE’s automated pore axis detection can produce suboptimal results for asymmetric or irregularly shaped pores, we defined the pore axis using a geometry-based procedure for each structure. Specifically, the β-barrel region was identified based on secondary structure annotations generated using the DSSP algorithm [78] as implemented in MDTraj (v1.10.3) [79]. Continuous β-strand segments containing at least 10 consecutive residues were required for pore-axis definition and downstream pore-geometry analysis. Models lacking sufficiently continuous β-strand architecture, including structurally incomplete β-ribbon or partially unfolded conformations, were excluded from pore-related analyses because reliable channel axes and lumen profiles could not be robustly defined. For each homomeric nanopore, we calculated both the geometric center of the entire structure and the centroid of the identified β-barrel region. The vector connecting these 2 points was used to define the pore axis (cvect). The β-barrel centroid was designated as the starting point of the HOLE scan and recorded as cpoint in Supplementary Table S7. Both cpoint coordinates and axis vectors were calculated from the membrane-oriented structures generated by PPM 3.0, ensuring consistency between membrane embedding and pore geometry analyses. To facilitate reproducibility, the cpoint coordinates and axis vectors used for every model are provided in Supplementary Table S7. This axis and origin were then provided as input to HOLE, which performed a scan in 0.1 Å steps along the defined axis until reaching the extracellular opening. To ensure robustness and mitigate sensitivity to termination thresholds, we repeated the analysis across a range of end distances (10–60 Å, in 2 Å intervals). The most consistent local minimum radius was selected as the representative constriction point for each nanopore.

## Additional files

**Supplementary Fig. S1**. Evaluation of conformational landscape and template independence in αHL-like models.

**Supplementary Fig. S2** Comparison of self-confidence scores between AeL prepore (purple) and final pore (teal) models.

**Supplementary Fig. S3** Conformational grouping of AeL-like models predicted by (A) AFM and (B) AF3.

**Supplementary Fig. S4** Comparison of multimer-level self-confidence scores (ipTM, pTM, and average pLDDT) for AeL models predicted by AF3 with and without PLM, respectively.

**Supplementary Fig. S5** Comparison of multimer-level self-confidence scores (ipTM, pTM, and average pLDDT) for AeL models predicted by AF3 with and without K+ and Cl− ions, respectively.

**Supplementary Fig. S6** Examples of nanopore models unable to embed correctly.

**Supplementary Fig. S7** Examples of nanopore protein models with tilt angle >10°.

**Supplementary Fig. S8**. Paired comparison of GPU inference time (top) and peak VRAM usage (bottom) for 50 representative CsgG-like nonameric complexes in their full-length (red) and trimmed (blue) states.

**Supplementary Fig. S9**. Quantitative validation of structural integrity and prediction confidence following sequence trimming.

**Supplementary Fig. S10**. Visualization of sequence trimming in representative CsgG-like models.

**Supplementary Fig. S11**. Violin plot of hydrophobic thickness (Å) for all nanopore candidates.

**Supplementary Fig. S12**. Distribution of mpDockQ scores and their relationship with AlphaFold-derived ipTM values.

**Supplementary Fig. S13**. The four representative nanopores for MD simulations.

**Supplementary Fig. S14**. Dynamical stability of representative nanopores during 100 ns MD simulations.

**Supplementary Fig. S15**. Dynamic pore radius profiles along the channel axis.

**Supplementary Fig. S16**. Examples of nanopore protein models with radius <1 Å or >12 Å.

**Supplementary Fig. S17**. Examples of switching conformation by adding PLM in co-folding prediction.

**Supplementary Table S1**. Experimentally resolved pore-like structures of representative nanopore types in the PDB.

**Supplementary Table S2**. Model counts of each step of the mining workflow (steps 1–5) and improved embedding in joint AF3-predictions with PLM molecules for incorrectly embedded AeL, MspA, and CsgG models.

**Supplementary Table S3**. Structural similarity (TM-score) of the experimental structures of AeL nanopores to reference conformations.

**Supplementary Table S4**. Structural similarity (TM-score) of the experimental structures of α-HL nanopores to reference conformations.

**Supplementary Table S5**. AF3 prediction results of AeL-like nanopores with ligands.

**Supplementary Table S6**. Taxonomic distribution of nanopore homologs.

**Supplementary Table S7**. The exact coordinates (cpoint) and axis vectors (cvect) used for each model.

**Supplementary Table S8**. Summary statistics of mpDockQ scores across nanopore families and conformational states.

## Supplementary Material

giag076_Supplemental_Files

giag076_Authors_Response_To_Reviewer_Comments_Original_Submission

giag076_GIGA-D-26-00030_Original_Submission

giag076_GIGA-D-26-00030_Revision_1

giag076_Reviewer_1_Report_Original_Submission Reviewer 1 -- 2/14/2026

giag076_Reviewer_1_Report_Revision_1 Reviewer 1 -- 6/7/2026

giag076_Reviewer_2_Report_Original_Submission Reviewer 2 -- 2/16/2026

giag076_Reviewer_2_Report_Revision_1 Reviewer 2 -- 5/26/2026

## Data Availability

The website of NanoporeDB is freely accessible at [80]. NanoporeDB has been registered in SciCrunch (RRID: SCR_028466). The protein nanopore mining and analysis workflow is available at WorkflowHub [81].
