# Supplementary material for: NanoporeDB: a structural resource of multimeric protein nanopores for single-molecule sensing
Source: Gigascience. 2026 Jun 25;15:giag076. doi: 10.1093/gigascience/giag076 (PMC13397529; doi:10.1093/gigascience/giag076)
Supplement: giag076_Supplemental_Files [file giag076_supplemental_files.zip › Supplementary materials_2026015.docx]

**Supplemental materials**

NanoporeDB: A Structural Resource Of Multimeric Protein Nanopores For Single-Molecule Sensing

Yuqian Liu (刘宇倩)^1,3^, Zidong Su (苏子栋)^3,8^, Wenzhen Yang (杨文真)^2^, Denghui Li (李登辉)^3,5^, Jiawen Zhang (章佳文)^2,4^, Yuning Zhang (张宇宁)^2,4^, Tao Zeng (曾涛)^2,4^, Yong Zhang (张勇)^6^, Yuxiang Li (黎宇翔)^6^, Guangyi Fan (范广益)^5,13^, Kailong Ma (麻凯龙)^2^, Shanshan Liu (刘姗姗)^7,14,15^, Xun Xu (徐讯)^1,2,10,11^, Yuliang Dong (董宇亮)^2,4,10,12,*^, Zongan Wang (王宗安)^2,3,9,10*^

^1^College of Life Sciences, University of Chinese Academy of Sciences, Beijing 100049, China

^2^BGI Research, Shenzhen 518083, China

^3^BGI Research, Sanya 572025, China

^4^BGI Hangzhou CycloneSEQ Technology Co., Ltd, Hangzhou 310030, China

^5^BGI Research, Qingdao 266555, China

^6^BGI Research, Wuhan 430074, China

^7^BGI, Shenzhen 518083, China

^8^School of Artificial Intelligence, University of Chinese Academy of Sciences, Beijing 100049, China

^9^Hainan Technology Innovation Center for Marine Biological Resources Utilization (Preparatory Period), BGI Research, Sanya 572025, China

^10^State Key Laboratory of Genome and Multi-omics Technologies, BGI Research, Shenzhen 518083, China

^11^Guangdong Provincial Key Laboratory of Genome Read and Write, BGI Research, Shenzhen 518083, China

^12^Shenzhen Engineering Laboratory for Molecular Enzymology, BGI Research, Shenzhen 518083, China

^13^Shenzhen Key Laboratory of Bioenergy, BGI Research, Shenzhen 518083, China

^14^Shenzhen Key Laboratory of Marine Biology Genomics, BGI Research, Shenzhen 518083, China

^15^Institution of Deep-Sea Life Sciences, IDSSE-BGI, Hainan Deep-sea Technology Laboratory, Sanya 572000, China

*Correspondence: [wangzongan@genomics.cn](mailto:wangzongan@genomics.cn), [dongyuliang@genomics.cn](mailto:dongyuliang@genomics.cn)

Lead contact: [wangzongan@genomics.cn](mailto:wangzongan@genomics.cn)


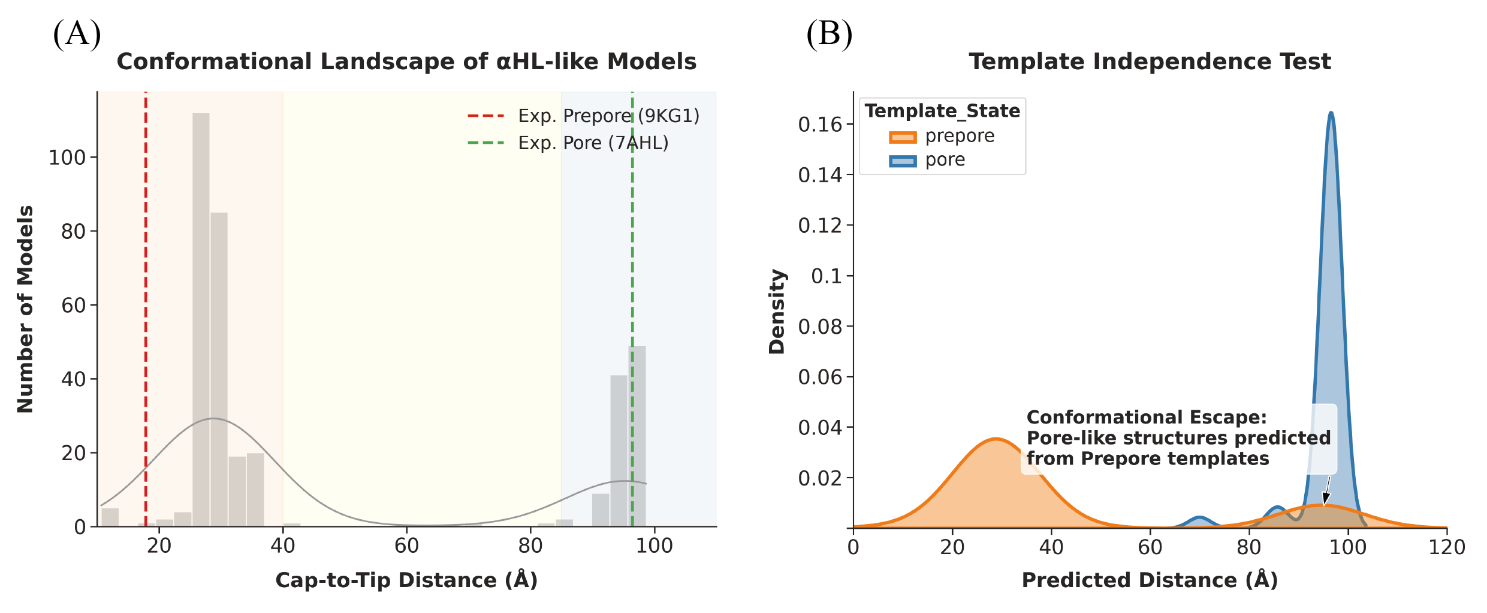
**Supplementary Fig. S1.** **Evaluation of conformational landscape and template independence in αHL-like models.** (A) The distribution of Cap-to-Tip distances reveals a distinct bimodal pattern, capturing both the folded prepore-like state and the extended pore-like state. Red and green dashed lines indicate reference distances from experimental prepore (PDB 9KG1) and pore (PDB 7AHL) structures, respectively. Shaded background colors delineate the structural classification zones: prepore-like (orange), late-prepore-like (yellow), and pore-like (blue). (B) Template independence test. Comparison of predicted distance distributions for candidates identified using prepore-state templates (orange) versus pore-state templates (blue). The substantial overlap between the two density curves and the presence of "conformational escape" (highlighted by the arrow, where prepore templates yielded pore-like predictions) demonstrate that the final structural outcomes are driven by intrinsic sequence properties rather than the conformational state of the initial search template.


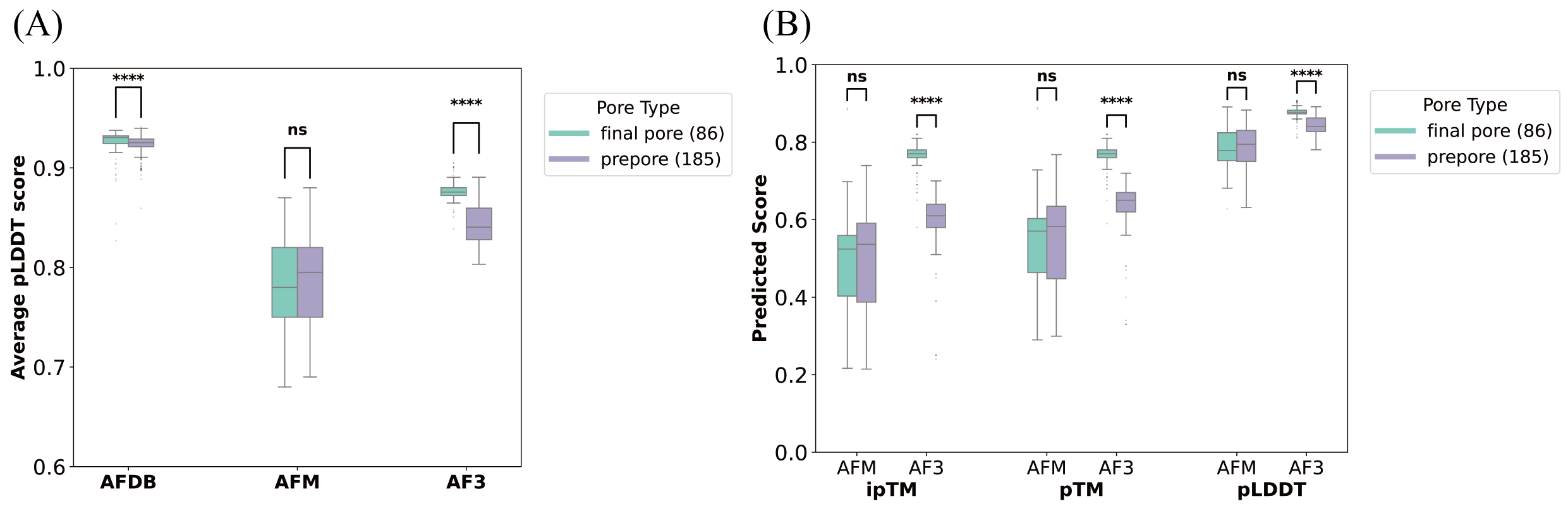


**Supplementary Fig. S2.** Comparison of self-confidence scores between AeL prepore (purple) and final pore (teal) models. (A) Monomer-level average pLDDT scores for AeL models from the AFDB, as well as those predicted by AFM and AF3. (B) Multimer-level scores (ipTM, pTM, and average pLDDT) of AFM and AF3 predictions. Significances of paired comparisons were calculated using two-sided Wilcoxon–Mann–Whitney U-test (*****P* < 0.0001).


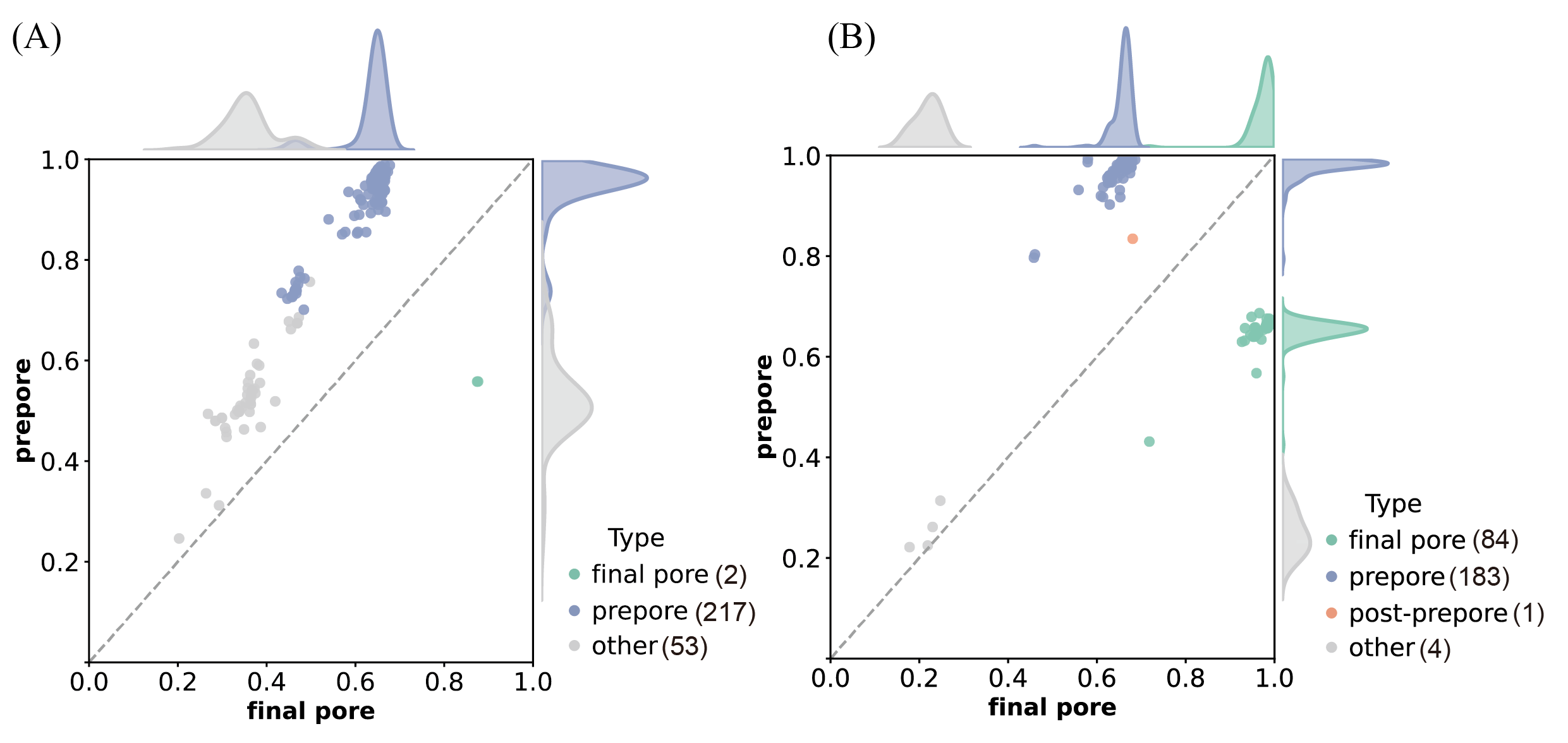


**Supplementary Fig. S3.** Conformational grouping of AeL-like models predicted by (A) AFM and (B) AF3**.** Scatter plots show TM-scores of predicted models relative to reference prepore (y-axis) and final pore (x-axis) structures. Points are colored by the assigned states. Models with TM-scores < 0.7 against the reference structures were classified as “other”.


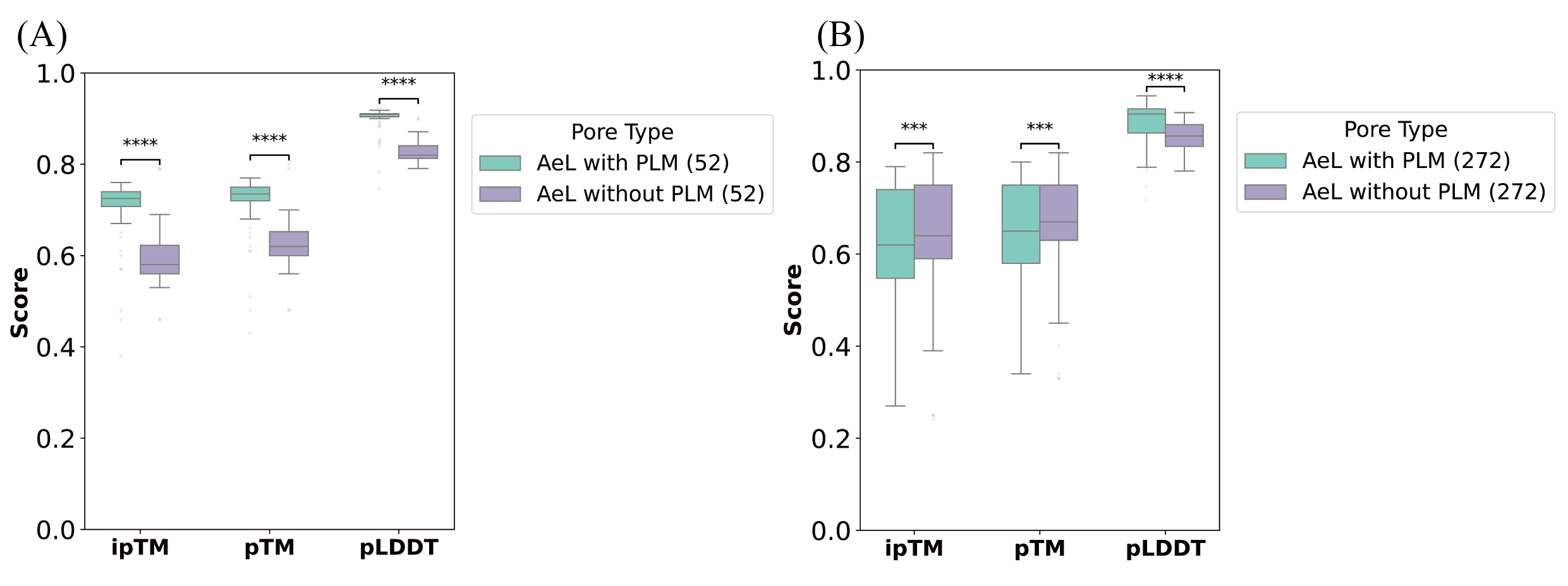


**Supplementary Fig. S4.** Comparison of multimer-level self-confidence scores (ipTM, pTM, and average pLDDT) for AeL models predicted by AF3 with and without PLM, respectively. (A) The subset of 52 models that switched conformations with addition of PLM molecules. (B) All 272 AF3 models. Significances of paired comparisons were calculated using two-sided Wilcoxon–Mann–Whitney U-test (****P* < 0.001, *****P* < 0.0001).


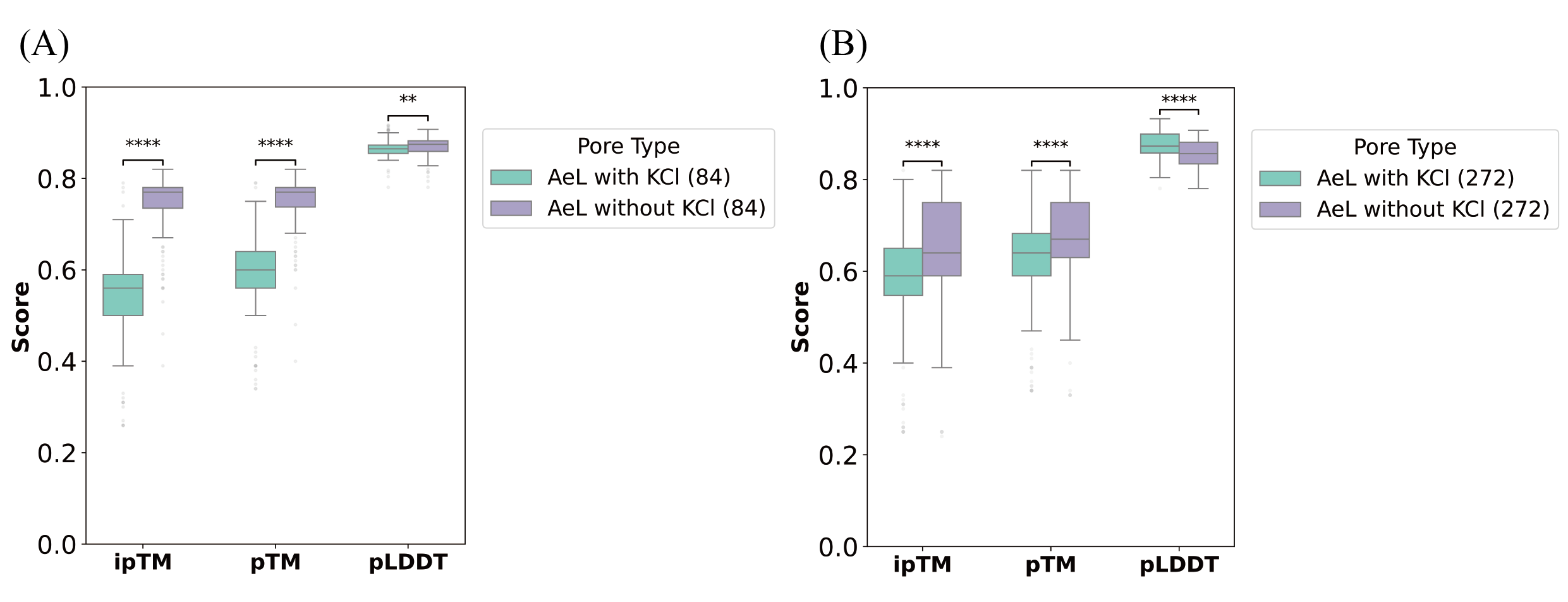


**Supplementary Fig. S5.** Comparison of multimer-level self-confidence scores (ipTM, pTM, and average pLDDT) for AeL models predicted by AF3 with and without K^+^ and Cl^-^ ions, respectively. (A) The subset of 84 models that switched conformations with addition of K^+^ and Cl^-^ ions. (B) All 272 AF3 models. Significances of paired comparisons were calculated using two-sided Wilcoxon–Mann–Whitney U-test (***P* < 0.01, *****P* < 0.0001).


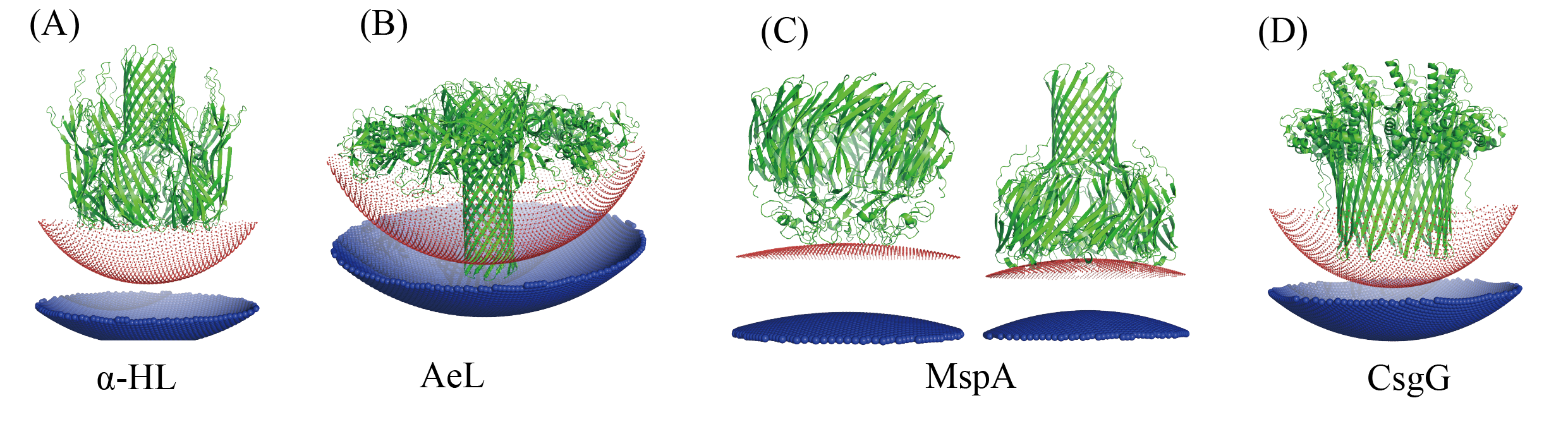


**Supplementary Fig. S6.** Examples of nanopore models unable to embed correctly. (A) α-HL (UniRef90 ID: UniRef90_UPI000D7CFDF9), (B) AeL (AFDB ID: A0A0T6TY63), (C) MspA (UniRef90 ID: UniRef90_A0A1D8T2P1, UniRef90 ID: UniRef90_UPI0027DB7C8D), and (D) CsgG (AFDB ID: A0A1T4SF25, AFDB ID: A0A4U2EGC6).


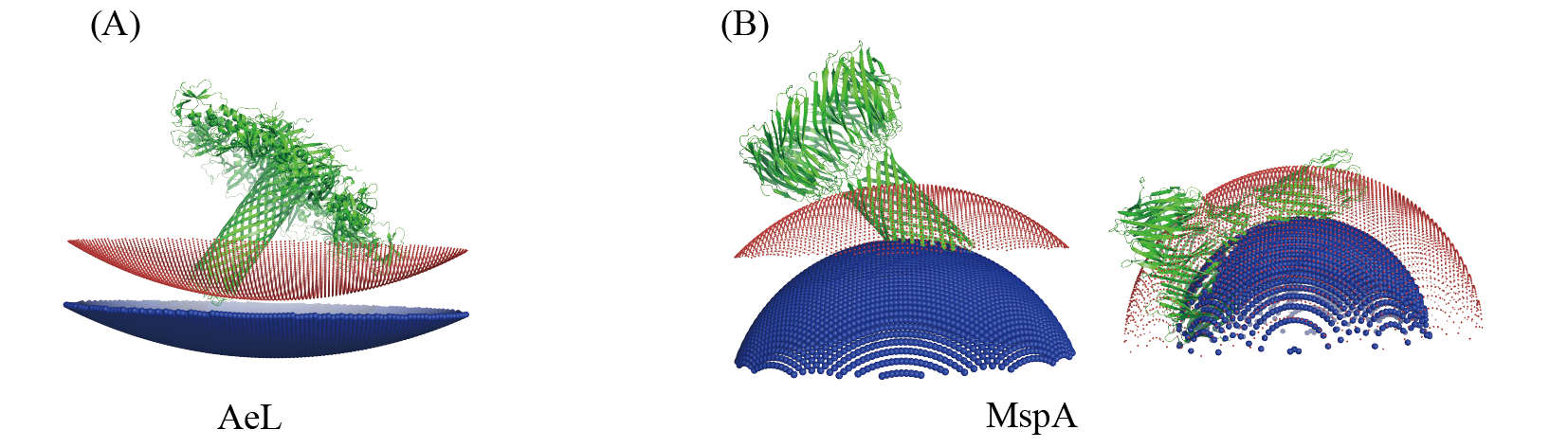


**Supplementary Fig. S7.** Examples of nanopore protein models with tilt angle > 10°. (A) AeL (UniRef90 ID: UniRef90_UPI00202CC0C0), (B) MspA (UniRef90 ID: UniRef90_UPI001CCE9F94, UniRef90 ID: UniRef90_UPI00326714A9).


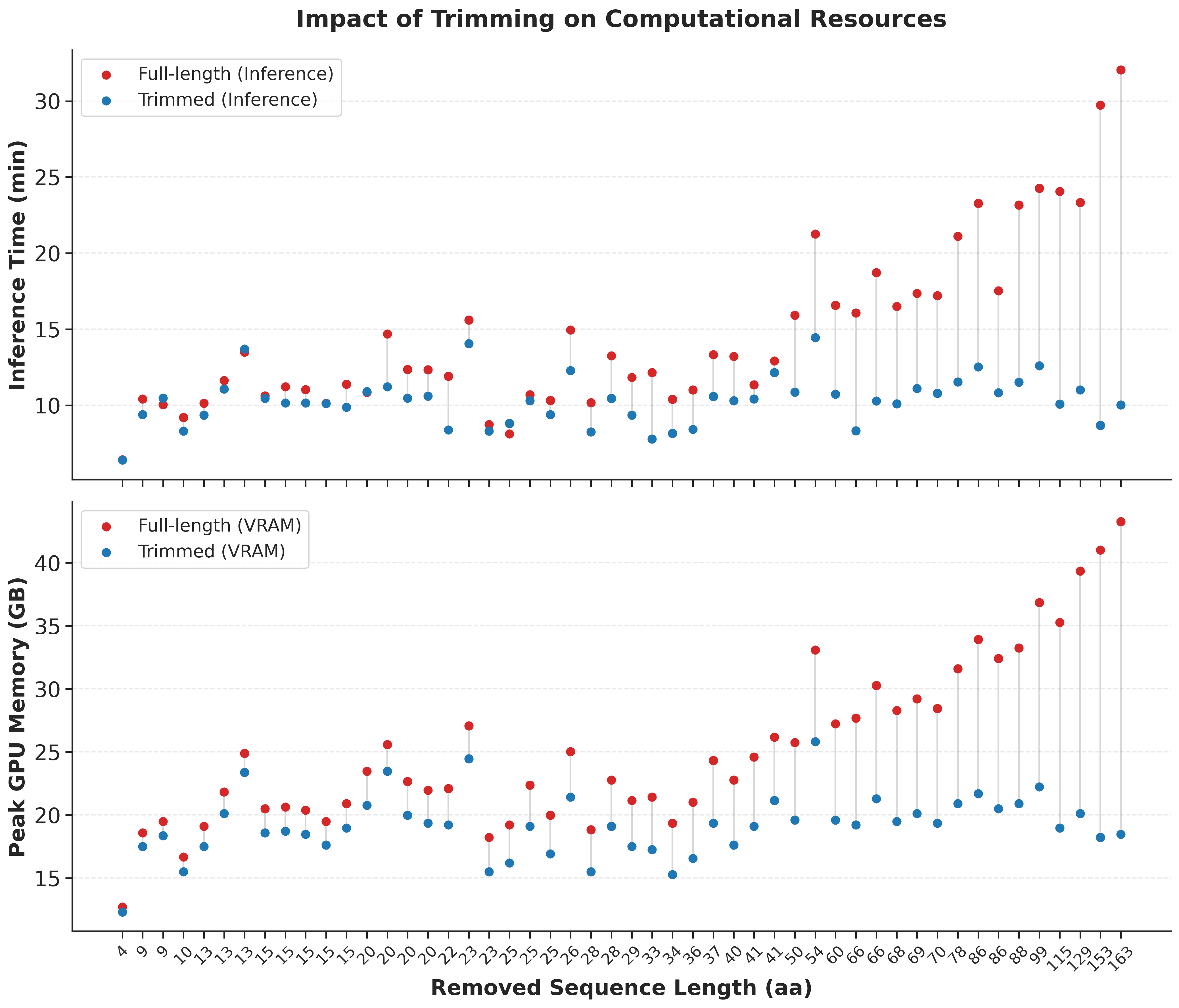


**Supplementary Fig. S8.** Paired comparison of GPU inference time (top) and peak VRAM usage (bottom) for 50 representative CsgG-like nonameric complexes in their full-length (red) and trimmed (blue) states. Data are sorted by the length of the removed sequence.


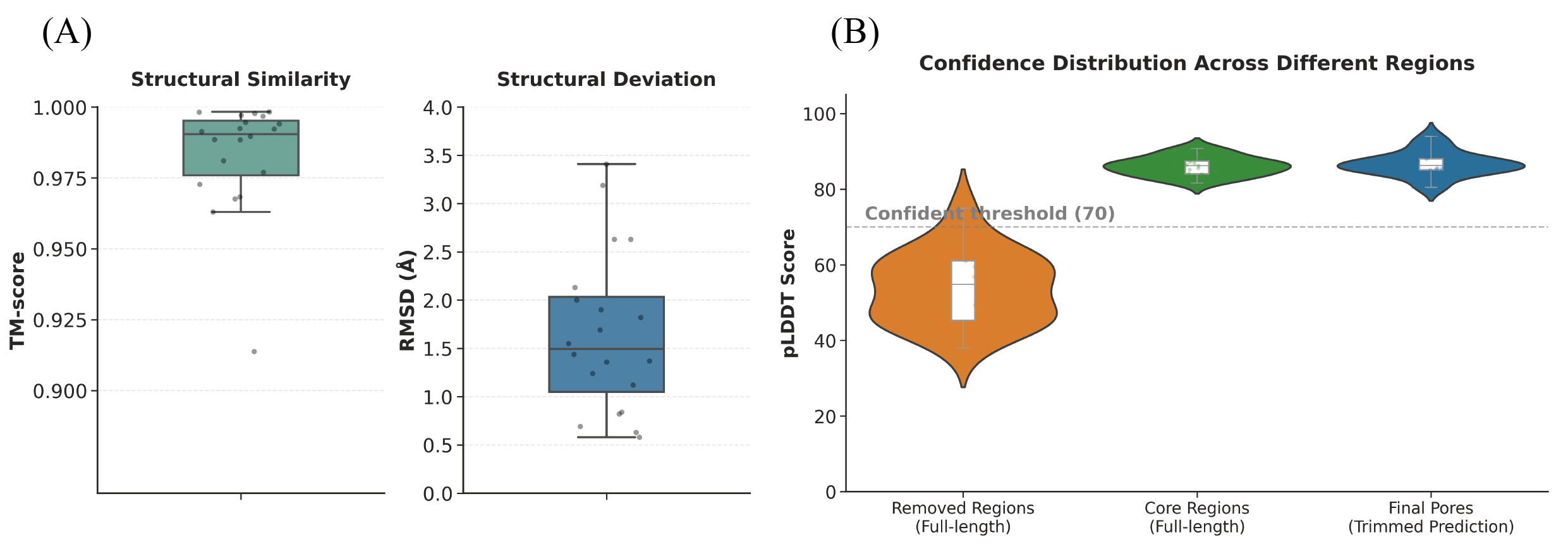


**Supplementary Fig. S9.** Quantitative validation of structural integrity and prediction confidence following sequence trimming. (A) Structural consistency between trimmed and full-length CsgG-like models (n = 20). Left: Boxplot of TM-scores (normalized by the length of the trimmed model), showing a median value > 0.98. Right: Boxplot of backbone RMSD (Å) calculated over the aligned pore-forming core. The high TM-scores and low RMSD values (~1.5 Å) demonstrate that the removal of terminal segments does not induce structural distortion in the functional nanopore architecture. (B) Confidence distribution (pLDDT) across different sequence regions. Violin plots compare the per-residue confidence for removed regions (orange), kept core regions within full-length models (green), and the final trimmed predictions used in the database (blue). The removed segments exhibit significantly lower confidence scores, characteristic of intrinsically disordered regions (IDRs), which justifies their exclusion to enhance the signal-to-noise ratio of the predicted functional core.

**Supplementary Fig. S10.** Visualization of sequence trimming in representative CsgG-like models. Structural superposition of trimmed models (blue) and full-length predictions (grey), with the removed segments highlighted in orange-red. (Left) AF-A0A329Y2Y4, (Right) UniRef90_UPI0018F0D173.


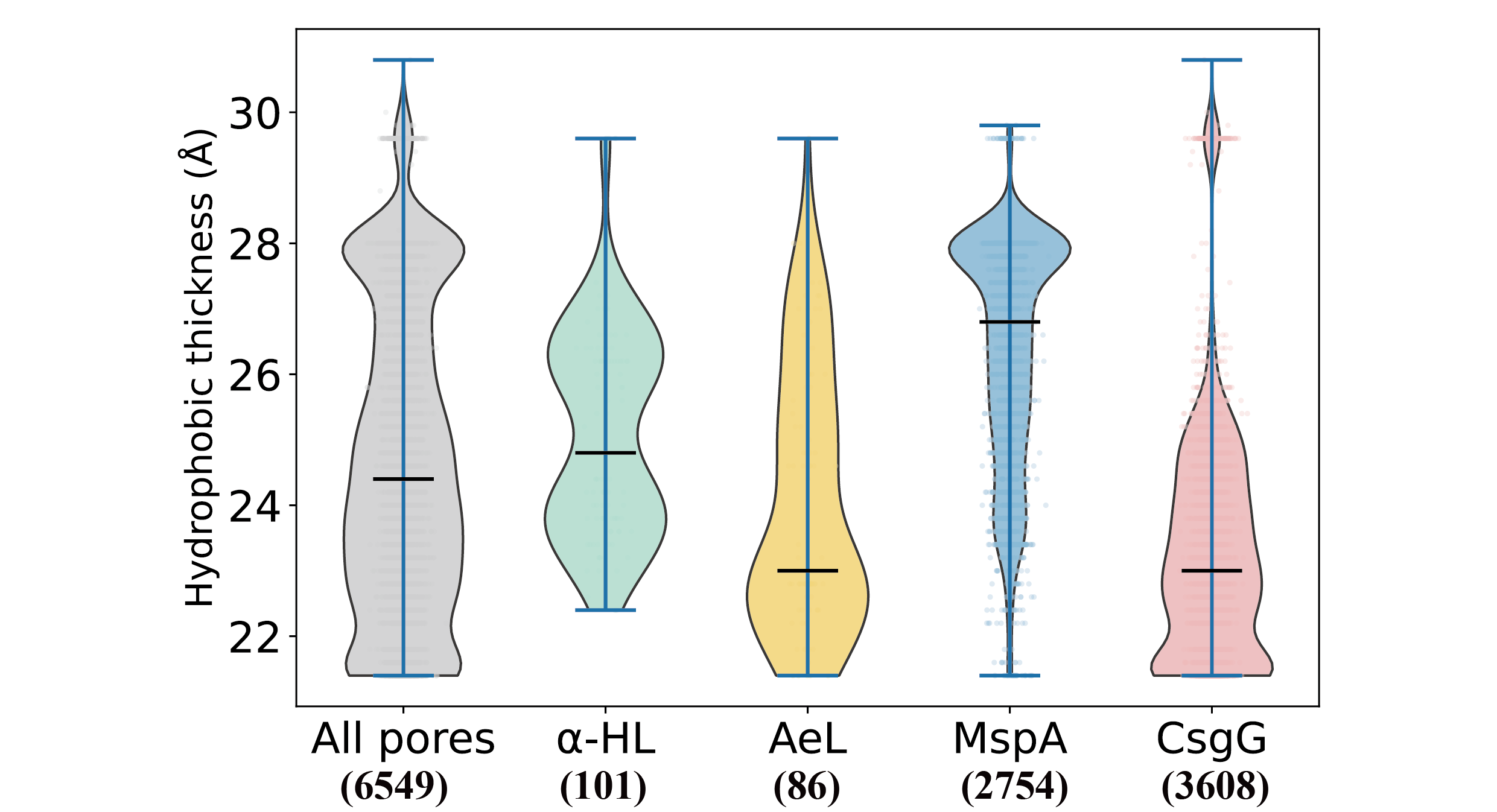


**Supplementary Fig. S11.** Violin plot of hydrophobic thickness (Å) for all nanopore candidates.


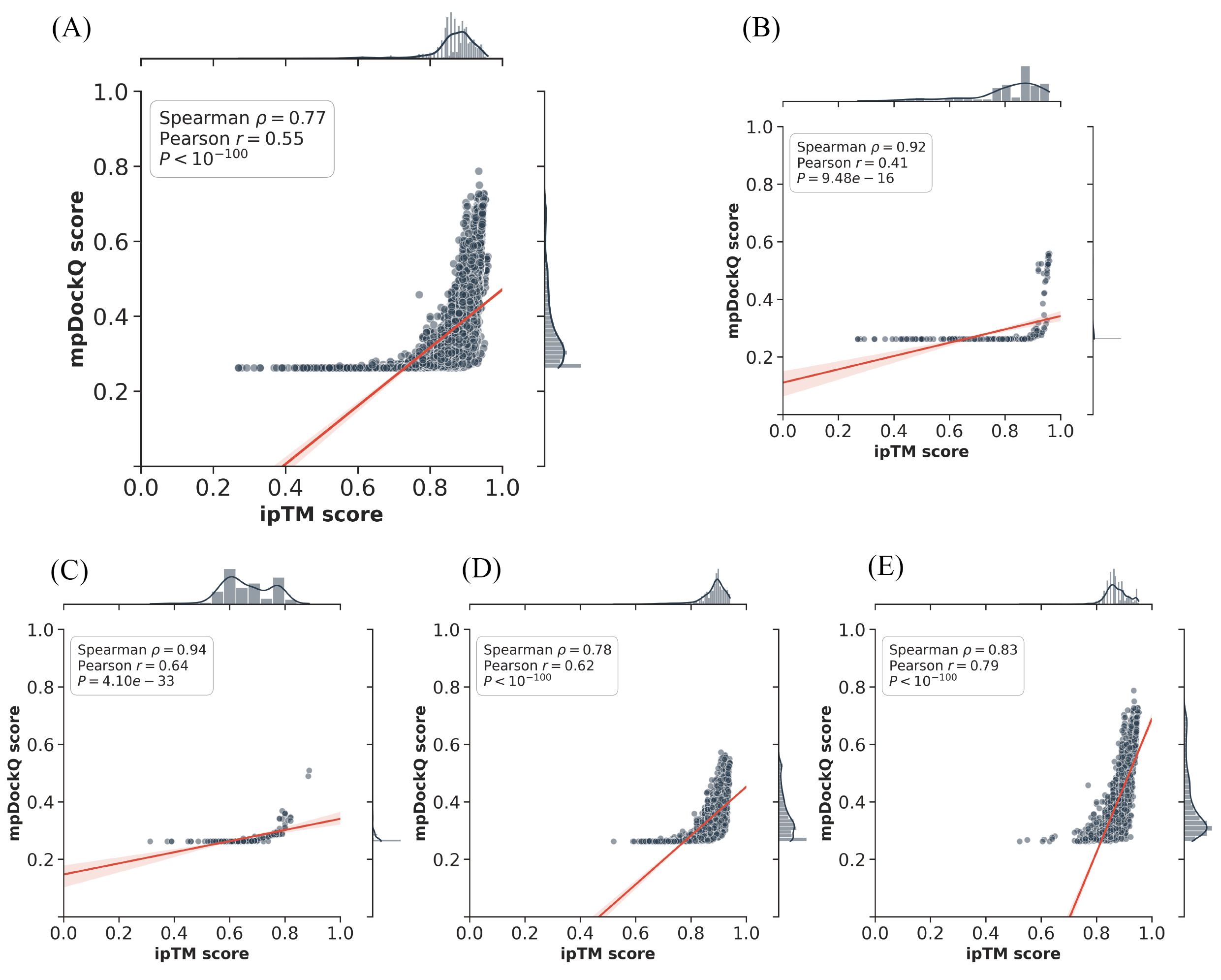


**Supplementary Fig. S12.** Distribution of mpDockQ scores and their relationship with AlphaFold-derived ipTM values. (A) All nanopore models, (B-E) α-HL, AeL, MspA, and CsgG nanopores, respectively. Scatter plots show the association between mpDockQ and ipTM scores. Marginal histograms and kernel density estimates illustrate the distribution of mpDockQ values within each dataset. Median and interquartile range (IQR) statistics for each family are summarized in Supplementary Table S8.

**Supplementary Fig. S13.** The four representative nanopores for MD simulations. Left to right: α-HL (UniRef90_UPI000BF7BC04), AeL (UniRef90_UPI000B8E5A08), MspA (MGYP001806187341), and CsgG (UniRef90_X1AGU0).


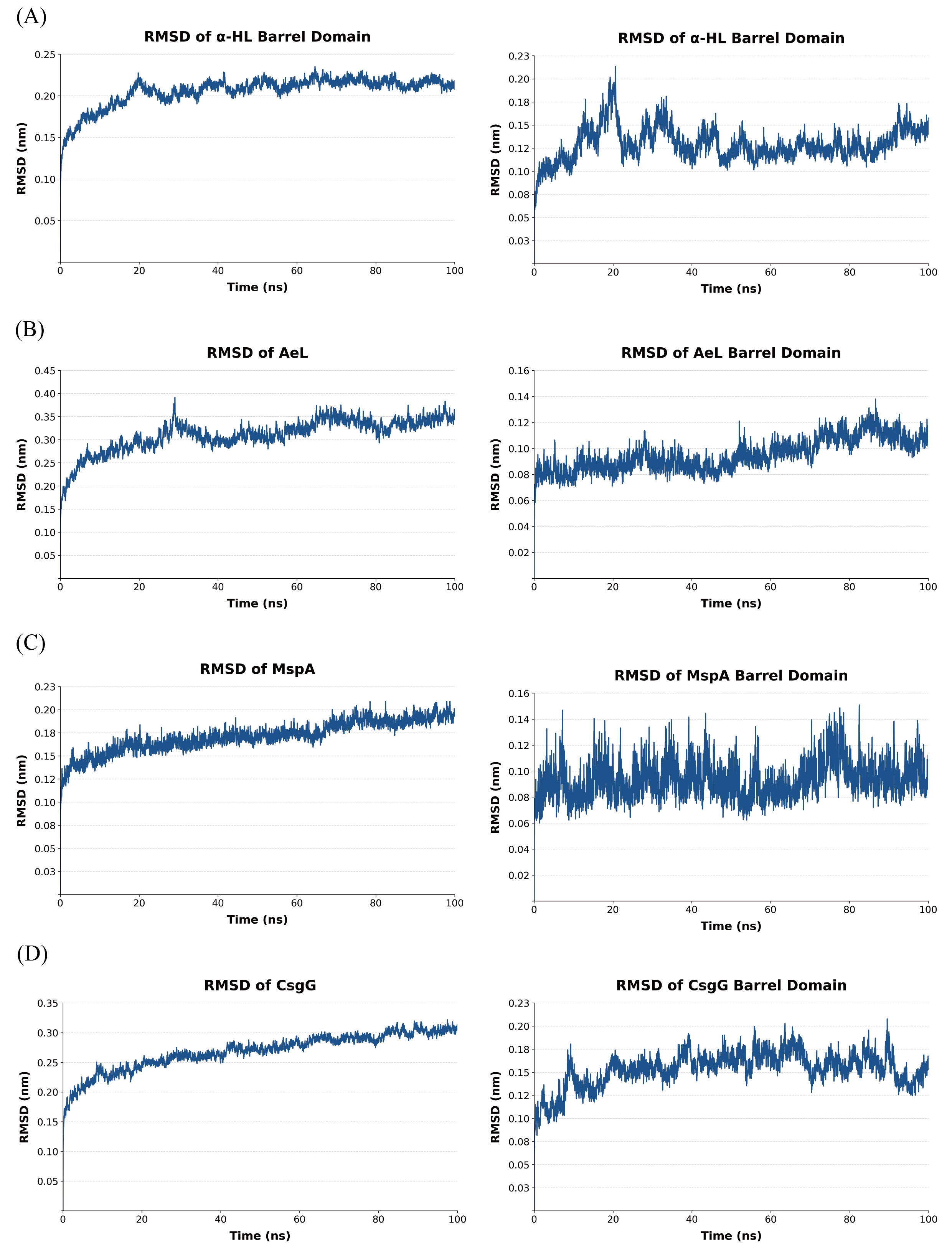


**Supplementary Fig. S14**. Dynamical stability of representative nanopores during 100 ns MD simulations. (A-D) RMSD curves for α-HL, AeL, MspA, and CsgG (IDs as specified in Fig. S13). For each panel, the left plot shows the RMSD of the entire protein complex, whereas the right plot shows the RMSD of the pore-forming β-barrel domain only.


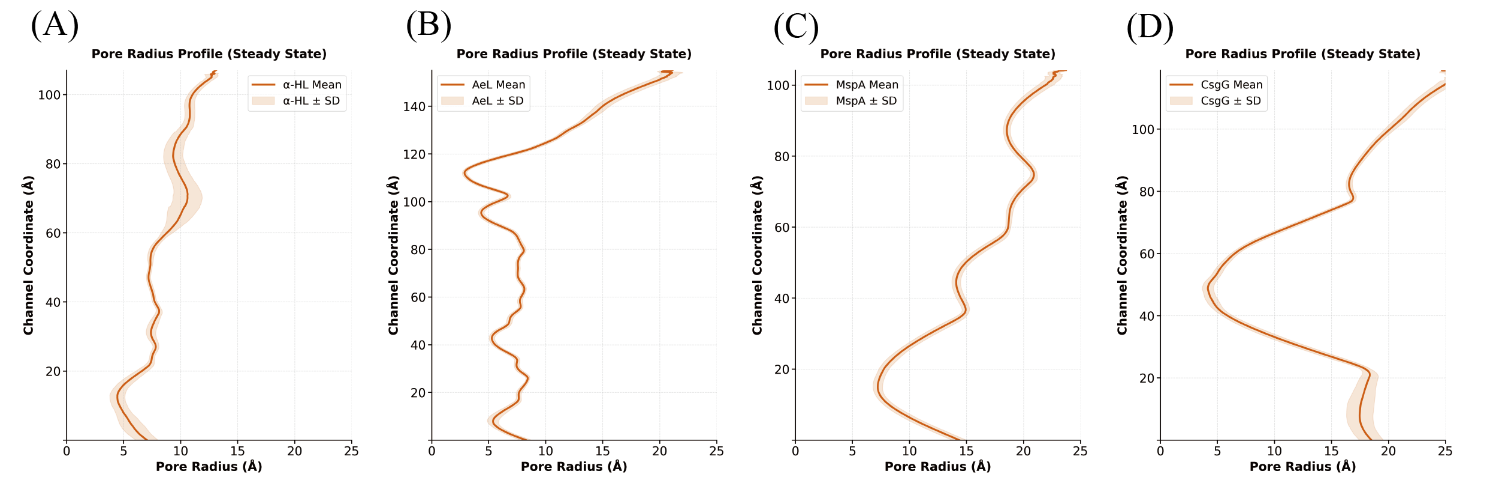


**Supplementary Fig. S15**. Dynamic pore radius profiles along the channel axis. (A-D) Pore radius profiles of representative α-HL, AeL, MspA, and CsgG nanopores, respectively (IDs as specified in Fig. S13). Radius profiles were calculated along the central pore axis using structures extracted from the last 50 frames of the 100 ns MD trajectories (one structure per frame).


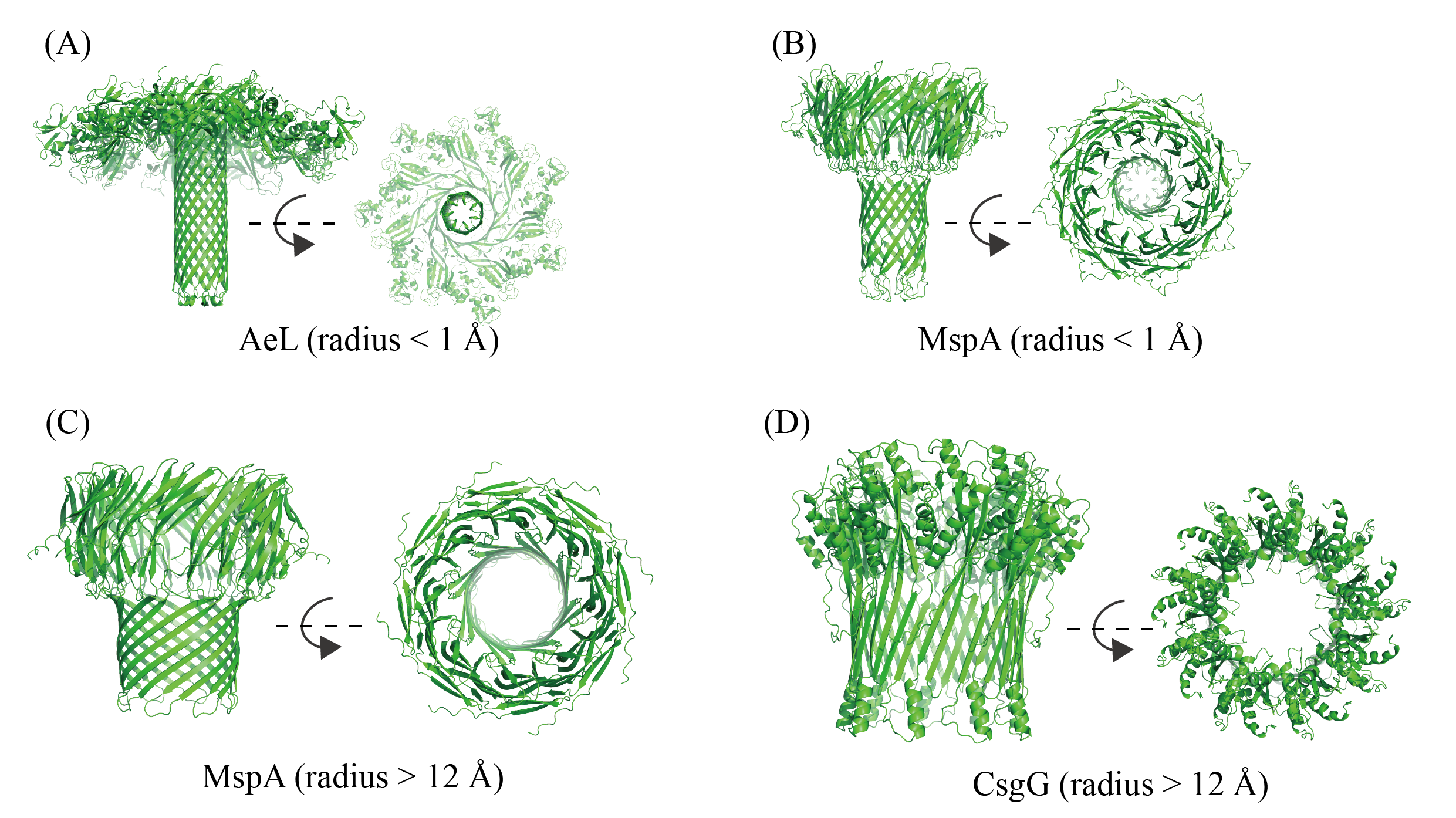


**Supplementary Fig. S16.** Examples of nanopore protein models with radius < 1 Å or > 12 Å. (A) AeL (radius = 0.98 Å, UniRef90 ID: UniRef90_A0A0F5VB76), (B) MspA (radius = 0.69 Å, AFDB ID: AF-A0A1A1WW01), (C) MspA (radius = 12.43 Å, UniRef90 ID: UniRef90_A0A3M2L262), and (D) CsgG (radius = 13.65 Å, AFDB ID: AF-A0A6M4GPJ4).


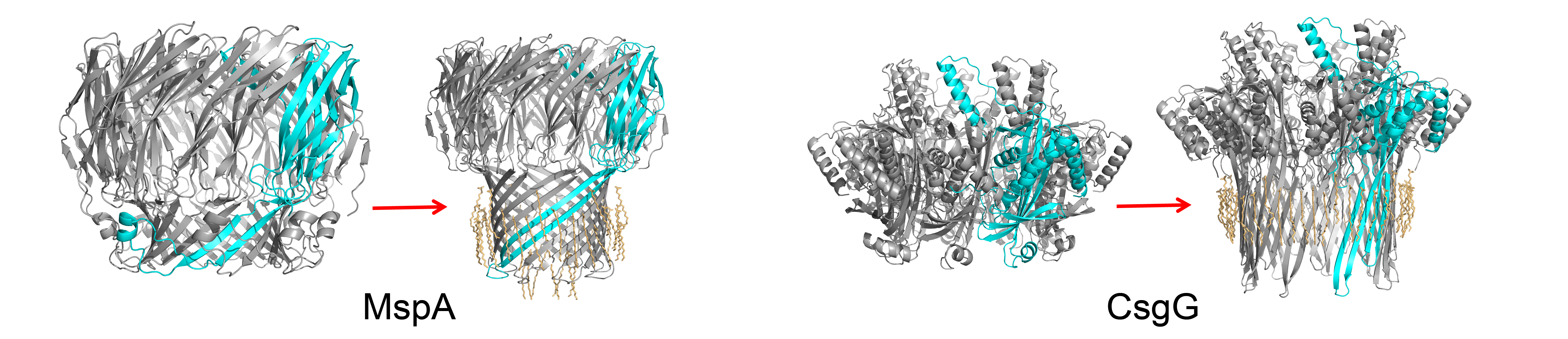


**Supplementary Fig. S17.** Examples of switching conformation by adding PLM in co-folding prediction of MspA (UniRef90 ID: UniRef90_A0A516WL96) and CsgG (AFDB ID: AF-A0A6N9RID5). The monomer chain of each protein is highlighted in cyan. PLM molecules are in orange.

**Supplementary Table S1.** Experimentally resolved pore-like structures of representative nanopore types in the PDB.

| Nanopore | PDB ID (total number of representative experimental structures) |
| --- | --- |
| α-HL | Prepore: 9KG1, 9KTM (2)  Late prepore: 9KG0, 9KTO (2)  Pore: 7AHL, 6U49, 3M2L, 3M3R, 7O1Q, 3M4E, 8JX2, 6U4P, 8JX3, 4P24, 3ANZ, 3M4D, 9KG3, 9KG6, 9KRE, 9KRF (16) |
| AeL | Prepore: 5JZH, 9FMX (2)  Post-prepore/quasipore: 5JZW (1)  Final pore: 5JZT, 6RB9, 9FNP, 9FNQ, 9FML, 9FM6 (6) |
| MspA | 1UUN (1) |
| CsgG | 6LQJ, 6L7C, 6LQH, 7BRM, 6L7A, 6SI7, 4UV3, 4Q79, 3X2R (9) |

**Supplementary Table S2.** Model counts of each step of the mining workflow (step 1 to 5) and improved embedding in joint AF3-predictions with PLM molecules for incorrectly embedded AeL, MspA, and CsgG models.

|  | α-HL | AeL | MspA | CsgG |
| --- | --- | --- | --- | --- |
| Step1: Name search | 998 | 548 | 1712 | 6036 |
| Step2: Structure-based search | 39 | 293 | 1364 | 1179 |
| Step3: Sequence-based search | 75 | 61 | 1726 | 2466 |
| Step4: Multimeric structure prediction | 103 | 353 | 3026 | 3618 |
| Step5: High-quality structure filtration (AFM : AF3) | 47 (43 : 4) | 272 (36 : 236) | 2754  (2293 : 461) | 3608  (1072 : 2536) |
| Models embedded correctly (AFM : AF3) | 46 (42 : 4) | 20 (0 : 20) ^1^ | 2663  (2229 : 434) | 3118  (1030 : 2088) |
| Models embedded incorrectly (AFM : AF3) | 1 (1 : 0) | 66 (2 : 64) | 91 (64 : 27) | 490 (42 : 448) |
| Models embedded incorrectly with ill-predicted TM region ^2^ (AFM : AF3) | N.A. | N.A. | 32 (21 : 11) | 41 (9 : 32) |
| Models able to embed correctly with PLM (AFM : AF3) | N.A. | 31 (0 : 31) ^3^ | 26 (18 : 8) ^4^ | 19 (1 : 18) ^4^ |

Note:

1. Only AeL models in the final pore state were subjected to membrane embedding analysis, including 2 AFM models and 84 AF3 models (Figure 2D right).

2. Models embedded incorrectly and exhibiting visible structural distortions in the transmembrane (TM) region.

3. 31 out of 66 incorrectly embedded AeL models were able to embed correctly in the joint AF3 prediction with PLM molecules.

4. 26 out of 32 incorrectly embedded MspA models with structural distortion in TM region and 19 out of 41 CsgG models were able to embed correctly in the joint AF3 prediction with PLM molecules.

**Supplementary Table S3.** Structural similarity (TM-score) of the experimental structures of AeL nanopores to reference conformations.

| PDB ID (Conformation) | Prepore | Post-prepore | Quasipore | Final pore |
| --- | --- | --- | --- | --- |
| 5JZH (Prepore) | 1 | 0.950 | 0.691 | 0.666 |
| 9FMX (Prepore) | 1 | 0.951 | 0.691 | 0.667 |
| 5JZW (Post-prepore) | 0.858 | 1 | 0.709 | 0.674 |
| 5JZW (Quasipore) | 0.655 | 0.745 | 1 | 0.947 |
| 5JZT (Final pore) | 0.620 | 0.702 | 0.955 | 1 |
| 6RB9 (Final pore) | 0.266 | 0.322 | 0.461 | 1 |
| 9FM6 (Final pore) | 0.635 | 0.713 | 0.959 | 1 |
| 9FML (Final pore) | 0.664 | 0.744 | 0.985 | 1 |
| 9FNP (Final pore) | 0.615 | 0.698 | 0.942 | 1 |
| 9FNQ (Final pore) | 0.627 | 0.710 | 0.957 | 1 |

Note: The prepore state of AeL has two experimental structures and the final pore states has six. The structure similarity of a structure to a given conformational state refers to the highest TM-score of that structure compared to all the structures belonging to the state.

**Supplementary Table S4.** Structural similarity (TM-score) of the experimental structures of α-HL nanopores to reference conformations.

| PDB ID (Conformation) | Prepore | Late prepore | Pore |
| --- | --- | --- | --- |
| 3ANZ (Pore) | 0.8041 | 0.9480 | 1 |
| 3M2L (Pore) | 0.8076 | 0.9521 | 1 |
| 3M3R (Pore) | 0.8074 | 0.9518 | 1 |
| 3M4D (Pore) | 0.8075 | 0.9521 | 1 |
| 3M4E (Pore) | 0.8076 | 0.9516 | 1 |
| 4P24 (Pore) | 0.8125 | 0.9517 | 1 |
| 6U49 (Pore) | 0.8083 | 0.9529 | 1 |
| 6U4P (Pore) | 0.8077 | 0.9528 | 1 |
| 7AHL (Pore) | 0.8088 | 0.9539 | 1 |
| 7O1Q (Pore) | 0.8103 | 0.9516 | 1 |
| 8JX2 (Pore) | 0.8092 | 0.9545 | 1 |
| 8JX3 (Pore) | 0.8091 | 0.9546 | 1 |
| 9KG3 (Pore) | 0.8085 | 0.9554 | 1 |
| 9KG6 (Pore) | 0.8087 | 0.9546 | 1 |
| 9KRE (Pore) | 0.8084 | 0.9548 | 1 |
| 9KRF (Pore) | 0.8082 | 0.9549 | 1 |
| 9KG1 (Prepore) | 1 | 0.9750 | 0.9777 |
| 9KTM (Prepore) | 1 | 0.9758 | 0.9782 |
| 9KG0 (Late prepore) | 0.8455 | 1 | 0.9997 |
| 9KTO (Late prepore) | 0.8454 | 1 | 0.9991 |

**Supplementary Table S5.** AF3 prediction results of AeL-like nanopores with ligands.

|  | AeL | AeL + PLM ^1^ | AeL + 10 K^+^ + 10 Cl^-^ |
| --- | --- | --- | --- |
| Prepore | 183 | 140 | 231 |
| Post-prepore | 1 | 0 | 0 |
| Quasipore | 0 | 0 | 0 |
| Final pore | 84 | 127 | 22 |
| Other | 4 | 5 | 19 |
| Conformational change^2^ |  | 52 in total  **prepore to final pore: 46**  prepore to other: 1  post-prepore to final pore: 1  final pore to prepore: 4 | 84 in total  prepore to final pore: 4  prepore to other: 13  post-prepore to prepore: 1  **final pore to prepore: 64**  final pore to other: 2 |
| Final pore able to embed | 20 | 68 in total  45 kept to be final pore  23 converted from prepore | N.A. |
| Final pore able to embed correctly | 20 | 65 in total  42 kept to be final pore  23 converted from prepore | N.A. |

Note:

1. Number of added PLM molecules were determined based the number of available tokens by AF3 online server and ranged from 36 to 54 (40 for 272 AeL predictions on average) (methods).

2. Number of conformational transitions were referenced to the results of predicting the protein nanopore alone.

**Supplementary Table S6.** Taxonomic distribution of nanopore homologs. Taxonomic annotations were obtained from AFDB and UniRef90. Bacterial, archaeal, and viral phylum are highlighted in blue, green, and orange, respectively. Entries without clear classification are labeled as Unclassified (grey).

| Nanopore Type | No. of PDB templates | Template source phylum | No. of mined homologs | Phylum distribution of mined homologs |
| --- | --- | --- | --- | --- |
| α-HL | 20 | Bacillota | 347 | Bacillota (346);  Unclassified (1) |
| AeL | 9 | Pseudomonadota (8); Bacillota (1) | 263 | Pseudomonadota (262);  Unclassified (1) |
| MspA | 1 | Actinomycetota | 2577 | Actinomycetota (2573); Bacteria_Unclassified (1);  Unclassified (3) |
| CsgG | 9 | Pseudomonadota | 1931 | Actinomycetota (2);  Aquificota (3);  Bacteroidota (9);  Bdellovibrionota (1);  Candidatus Azambacteria (1);  Chlorobiota (2);  Myxococcota (3);  Nitrospinota (2);  Nitrospirota (4);  Planctomycetota (2);  Pseudomonadota (1849);  Thermodesulfobacteriota (10);  Thermosulfidibacterota (1);  Bacteria_Unclassified (9);  Candidatus Thermoplasmatota (3);  Archaea_Unclassified (1);  Uroviricota (13);  Viruses_Unclassified (5);  Unclassified (11) |

**Supplementary Table S8.** Summary statistics of mpDockQ scores across nanopore families and conformational states. Median, first quartile (Q1), third quartile (Q3), and interquartile range (IQR) are reported for each group.

| Nanopore family (state) | Number of models | Median mpDockQ | IQR (Q3 - Q1) |
| --- | --- | --- | --- |
| α-HL (All) | 352 | 0.2625 | 0.0099 |
| α-HL (Pore) | 101 | 0.2925 | 0.2433 |
| AeL (All) | 272 | 0.2626 | 0.0219 |
| AeL (Pore) | 86 | 0.2882 | 0.0098 |
| MspA (All) | 2754 | 0.3283 | 0.0920 |
| CsgG (All) | 3608 | 0.3442 | 0.1399 |
| All_data (All) | 6986 | 0.3299 | 0.1118 |

**Supplementary Note 1. Impact of sequence trimming on membrane embedding and structural integrity.**

To assess the impact of sequence trimming on membrane embedding, we performed additional analyses focusing on CsgG-like proteins. First, among 3608 predicted CsgG-like models, 2918 were generated using trimmed sequences, of which 2493 (85.4%) showed correct membrane embedding, while 425 exhibited anomalous insertion. To determine whether these anomalies were caused by truncation, we re-predicted all 425 cases using full-length sequences with AF3. Among these, 339 (~80%) recovered correct membrane embedding, indicating that the absence of terminal regions—likely containing membrane-anchoring elements—contributes to the observed deviations. Second, to evaluate structural integrity, we compared trimmed and full-length predictions for 20 representative proteins. The resulting structures showed high similarity, with a mean RMSD of ~1.5 Å, demonstrating that trimming does not affect the pore-forming core architecture. Analysis of pLDDT distributions further showed that removed regions predominantly correspond to low-confidence or disordered segments. Finally, benchmarking on 50 representative proteins demonstrated that both inference time and GPU memory usage increase substantially with sequence length, supporting the necessity of trimming for large-scale prediction. Together, these results indicate that sequence trimming primarily affects membrane positioning in a subset of cases, but has minimal impact on the structural integrity of nanopore cores. Among the 425 anomalous cases, 339 recovered correct membrane embedding after full-length prediction. The remaining 86 models continued to exhibit atypical membrane positioning. Nevertheless, structural comparison showed that their pore-forming cores remained highly similar to the corresponding full-length predictions (mean RMSD ~1.5 Å). Therefore, these models were retained in NanoporeDB and annotated as having atypical membrane embedding, indicating that membrane-related parameters should be interpreted with caution.
