# Supplementary material for: NanoporeDB: a structural resource of multimeric protein nanopores for single-molecule sensing
Source: Gigascience. 2026 Jun 25;15:giag076. doi: 10.1093/gigascience/giag076 (PMC13397529; doi:10.1093/gigascience/giag076)
Supplement: giag076_Authors_Response_To_Reviewer_Comments_Original_Submission [file giag076_authors_response_to_reviewer_comments_original_submission.pdf]

Author's Response To Reviewer Comments

Dear Dr. Mu,
Thank you for your decision letter dated 2026-02-24 regarding our manuscript GIGA-D-26-00030. We also thank the reviewers for their constructive comments, which have helped us significantly improve the paper.

We have now completed the revision process, which took approximately three months as we performed substantial additional analyses to address all the points raised.

All changes have been marked in the revised manuscript using orange (for Reviewer #1) and blue (for Reviewer #2) text. A detailed point-by-point response to all reviewer comments is attached as "Response to referees.docx". We have also updated the supplementary data accordingly (including a separate file for supplementary Table S7).

We believe that the revised manuscript is now substantially strengthened and suitable for publication in GigaScience. We have also registered the necessary identifiers (RRID and workflow DOI) as requested, and these have been included in the manuscript.

We look forward to your final decision. Please do not hesitate to contact us if any further modifications are needed.

Sincerely yours,
Wang Zongan
On behalf of all authors

Editorial Manager®

Cookie Preference Center

We use cookies which are necessary to make our site work. We may also use additional cookies to analyze, improve and personalize our content and your digital experience. For more information, see our Cookie Policy.

You may choose not to allow some types of cookies. However, blocking some types may impact your experience of our site and the services we are able to offer. See the different category headings below to find out more or change your settings.

Allow all
Manage Consent Preferences

Strictly Necessary Cookies

Always active

These cookies are necessary for the website to function and cannot be switched off in our systems. They are usually only set in response to actions made by you which amount to a request for services, such as setting your privacy preferences, logging in or filling in forms. You can set your browser to block or alert you about these cookies, but some parts of the site will not then work.

Functional Cookies

These cookies enable the website to provide enhanced functionality and personalisation. They may be set by us or by third party providers whose services we have added to our pages. If you do not allow these cookies then some or all of these services may not function properly.

Performance Cookies

These cookies allow us to count visits and traffic sources so we can measure and improve the performance of our site. They help us to know which pages are the most and least popular and see how visitors move around the site.

Targeting Cookies

These cookies may be set through our site by our advertising partners. They may be used by those companies to build a profile of your interests and show you relevant adverts on other sites. If you do not allow these cookies, you will experience less targeted advertising.

Cookie List

Clear

Apply Cancel

Consent Leg.Interest

Confirm my choices

Power
