# Supplementary material for: NanoporeDB: a structural resource of multimeric protein nanopores for single-molecule sensing
Source: Gigascience. 2026 Jun 25;15:giag076. doi: 10.1093/gigascience/giag076 (PMC13397529; doi:10.1093/gigascience/giag076)
Supplement: giag076_Reviewer_2_Report_Original_Submission — Reviewer 2 -- 2/16/2026 [file giag076_reviewer_2_report_original_submission.pdf]

Reviewer Report

Title: NanoporeDB: A Structural Resource Of Multimeric Protein Nanopores For Single-Molecule Sensing

Version: Original Submission **Date:** 2/16/2026

Reviewer name: Reviewer 2

Reviewer Comments to Author:

This manuscript presents NanoporeDB, a structural resource of multimeric protein nanopores constructed using structure- and sequence-guided mining combined with AlphaFold-Multimer and AlphaFold3 predictions. The work is timely and potentially valuable for the nanopore community. In particular, the focus on multimeric assemblies, membrane-embedding analysis, and pore-geometry annotation represents a meaningful contribution. However, several methodological assumptions and interpretations require clarification or further discussion to ensure robustness and reproducibility. My comments are listed below.

1. One of the concerns is template dependence in the structural mining workflow. The structural mining strategy relies heavily on PDB-derived templates. While this approach is reasonable, it may introduce bias in the search space. The observed taxonomic concentration of certain nanopore families could partly reflect template-driven constraints rather than biological distribution. A brief discussion or sensitivity analysis regarding template dependence would strengthen the conclusions.
2. Interpretation of prepore dominance in AeL-like models is needed. The predominance of prepore-like conformations among AeL candidates is interesting. However, prepore structures were included during the structure-based search stage, which may naturally favor similar conformations. The possibility of template-induced bias should be discussed more explicitly. In this context, incorporating recently resolved Î±HL prepore structures (e.g., PDB 9KG1) into the same pipeline could provide an informative comparison. Such analysis may reinforce the interpretation without requiring additional experiments.
3. Structural filtering versus biological relevance should be considered. The exclusion of prepore-like Î±HL models at later pipeline stages raises a conceptual issue. Since prepore states are experimentally established, clarification is needed on how the TM-score threshold relates to functional relevance. A short explanation of the filtering rationale would be helpful.
4. Sequence trimming and membrane embedding consistency are important. For CsgG-like models, membrane embedding anomalies are attributed to the absence of native terminal regions, including membrane anchors. This observation is important, but it also highlights potential inconsistencies with the trimming strategy. Given the known functional role of terminal segments in membrane proteins, the trade-off between computational efficiency and structural validity should be discussed.
5. Reproducibility of the HOLE axis definition should be checked. The manual definition of pore axes is understandable, especially for asymmetric structures. However, reproducibility is a critical concern. Providing axis vectors or reference coordinates for each model would significantly improve the transparency and usability of the database.
6. Justification of structural quality thresholds is needed. Thresholds such as pLDDT â‰¥ 70 and TM-

score â‰¥ 0.8 appear reasonable, yet their selection criteria are not fully described. A concise rationale or reference to established practices would improve methodological clarity.
7. Selecting predicted structures based on alignment to known PDB assemblies may inadvertently suppress structurally novel candidates. Since the database aims to expand the nanopore repertoire, potential effects of this selection strategy merit discussion.
8. There are inconsistent qtmscore thresholds. The use of qtmscore â‰¥ 0.7 for AeL conformation assignment differs from other structural thresholds. The reasoning behind this choice should be clarified for consistency.
Minor Comments
9. The handling criteria for structurally incomplete Î²-strand or Î²-ribbon models could be clarified.
10. A brief note on potential version dependence of PPM predictions may be useful.
11. The term "locally optimized implementation" for AFM should be briefly specified.

Methods

Are the methods appropriate to the aims of the study, are they well described, and are necessary controls included? Choose an item.

Conclusions

Are the conclusions adequately supported by the data shown? Choose an item.

Reporting Standards

Does the manuscript adhere to the journal's guidelines on minimum standards of reporting? Choose an item.

Choose an item.

Statistics

Are you able to assess all statistics in the manuscript, including the appropriateness of statistical tests used? Choose an item.

Quality of Written English

Please indicate the quality of language in the manuscript: Choose an item.

Declaration of Competing Interests

Please complete a declaration of competing interests, considering the following questions:

- Have you in the past five years received reimbursements, fees, funding, or salary from an organisation that may in any way gain or lose financially from the publication of this manuscript, either now or in the future?
- Do you hold any stocks or shares in an organisation that may in any way gain or lose financially from the publication of this manuscript, either now or in the future?
- Do you hold or are you currently applying for any patents relating to the content of the manuscript?
- Have you received reimbursements, fees, funding, or salary from an organization that holds or has applied for patents relating to the content of the manuscript?
- Do you have any other financial competing interests?
- Do you have any non-financial competing interests in relation to this paper?

If you can answer no to all of the above, write 'I declare that I have no competing interests' below. If your reply is yes to any, please give details below.

There is no competing interests.

I agree to the open peer review policy of the journal. I understand that my name will be included on my report to the authors and, if the manuscript is accepted for publication, my named report including any attachments I upload will be posted on the website along with the authors' responses. I agree for my report to be made available under an Open Access Creative Commons CC-BY license (http://creativecommons.org/licenses/by/4.0/). I understand that any comments which I do not wish to be included in my named report can be included as confidential comments to the editors, which will not be published.

I agree to the open peer review policy of the journal

To further support our reviewers, we have joined with Publons, where you can gain additional credit to further highlight your hard work (see: https://publons.com/journal/530/gigascience). On publication of this paper, your review will be automatically added to Publons, you can then choose whether or not to claim your Publons credit. I understand this statement.

Yes Choose an item.
