# Supplementary material for: NanoporeDB: a structural resource of multimeric protein nanopores for single-molecule sensing
Source: Gigascience. 2026 Jun 25;15:giag076. doi: 10.1093/gigascience/giag076 (PMC13397529; doi:10.1093/gigascience/giag076)
Supplement: giag076_Reviewer_2_Report_Revision_1 — Reviewer 2 -- 5/26/2026 [file giag076_reviewer_2_report_revision_1.pdf]

Reviewer Report

Title: NanoporeDB: A Structural Resource Of Multimeric Protein Nanopores For Single-Molecule Sensing

Version: Revision 1 **Date:** 5/26/2026

Reviewer name: Reviewer 2

Reviewer Comments to Author:

The revised manuscript has been substantially improved, and the authors have addressed the major concerns raised in the previous review. In particular, the additional analyses regarding template dependence, conformational bias, sequence trimming, and structural reproducibility considerably strengthen the methodological rigor and robustness of the resource. The newly added supplementary analyses and controls are helpful and improve the overall clarity of the study. I only have a few minor comments that may further improve the manuscript.

1. The inclusion of axis vectors and cpoint coordinates in Supplementary Table S7 substantially improves reproducibility of the HOLE-based pore geometry analysis. However, it would further improve clarity if the Methods explicitly define the geometric meaning of cpoint (e.g., Î²-barrel centroid versus HOLE starting point) and clarify whether the coordinates correspond to structures after PPM orientation/membrane embedding.
2. The clarification regarding membrane slab representation in the response was helpful. In particular, the explanation that the apparent membrane intrusion into the lumen originates from the continuous slab representation used in PPM/OPM, rather than physical lipid penetration, resolved the previous concern. It may nevertheless be beneficial to include a concise explanation in the Methods section to avoid ambiguity for future readers.
3. There is a minor grammatical issue in the membrane embedding section:
"because the prepore state should not entered the membrane"
should be revised to
"because the prepore state should not enter the membrane".

Methods

Are the methods appropriate to the aims of the study, are they well described, and are necessary controls included? Choose an item.

Conclusions

Are the conclusions adequately supported by the data shown? Choose an item.

Reporting Standards

Does the manuscript adhere to the journal's guidelines on minimum standards of reporting? Choose an item.

Choose an item.

Statistics

Are you able to assess all statistics in the manuscript, including the appropriateness of statistical tests used? Choose an item.

Quality of Written English

Please indicate the quality of language in the manuscript: Choose an item.

Declaration of Competing Interests

Please complete a declaration of competing interests, considering the following questions:

- Have you in the past five years received reimbursements, fees, funding, or salary from an organisation that may in any way gain or lose financially from the publication of this manuscript, either now or in the future?
- Do you hold any stocks or shares in an organisation that may in any way gain or lose financially from the publication of this manuscript, either now or in the future?
- Do you hold or are you currently applying for any patents relating to the content of the manuscript?
- Have you received reimbursements, fees, funding, or salary from an organization that holds or has applied for patents relating to the content of the manuscript?
- Do you have any other financial competing interests?
- Do you have any non-financial competing interests in relation to this paper?

If you can answer no to all of the above, write 'I declare that I have no competing interests' below. If your reply is yes to any, please give details below.

No competing intereest.

I agree to the open peer review policy of the journal. I understand that my name will be included on my report to the authors and, if the manuscript is accepted for publication, my named report including any attachments I upload will be posted on the website along with the authors' responses. I agree for my report to be made available under an Open Access Creative Commons CC-BY license (http://creativecommons.org/licenses/by/4.0/). I understand that any comments which I do not wish to be included in my named report can be included as confidential comments to the editors, which will not be published.

I agree to the open peer review policy of the journal

To further support our reviewers, we have joined with Publons, where you can gain additional credit to further highlight your hard work (see: https://publons.com/journal/530/gigascience). On publication of this paper, your review will be automatically added to Publons, you can then choose whether or not to claim your Publons credit. I understand this statement.

Yes Choose an item.
